# Surface morphology effects on droplet spreading and rebound dynamics on subcooled superhydrophobic surfaces

**DOI:** 10.1038/s41598-025-14634-4

**Published:** 2025-08-12

**Authors:** Matic Može, Yuheng Shang, Samo Jereb, Nina Kovač, Miha Štucin, Tim Štrus, Peter Rodič, Matevž Zupančič, Maria Rosaria Vetrano, Iztok Golobič

**Affiliations:** 1 Faculty of Mechanical Engineering, University of Ljubljana, Aškerčeva c. 6, SI-1000 Ljubljana, Slovenia; 2 Department of Mechanical Engineering, Division of Applied Mechanics and Energy Conversion (TME), KU Leuven, B-3001 Leuven, Belgium; 3 Department of Physical and Organic Chemistry, Jožef Stefan Institute, Jamova c. 39, SI-1000 Ljubljana, Slovenia; 4 Jožef Stefan International Postgraduate School, Jamova c. 39, SI-1000 Ljubljana, Slovenia

**Keywords:** Droplet impact, Droplet rebound, Contact time, Spreading factor, Superhydrophobic surfaces, Icephobicity, Laser-textured surfaces, Mechanical engineering, Applied physics, Surface chemistry

## Abstract

Subcooled superhydrophobic surfaces have notable applications in aerospace, energy, and refrigeration industries. Superhydrophobic behavior can be achieved with different microscale surface morphologies which can impact the water repellency and icephobicity of the surface. To comprehensively study how surface microstructure influences the spreading, rebounding, and freezing behavior of impacting droplets at various surface temperatures and droplet velocities, several types of surfaces were prepared within this study. Specifically, the effect of structure depth (approx. 3–30 μm) and of the structure type (randomized structure or directional microchannels) was investigated by preparing deep and shallow laser-made structures with either stochastic or deterministic features. Droplet impact tests were performed at Weber numbers between 50 and 185 and across surface temperatures from 25 °C to -30 °C. Surface morphology had a minimal effect on the maximum spreading factor, which was otherwise found to decrease by up to 9.5% when reducing the surface temperature from 25 °C to -30 °C. High-speed imaging revealed that the poorest rebound performance across all surfaces occurred at We ≅ 120, where the transition from the regular rebound to the splashing regime led to a higher prevalence of partial rebounds or full adhesion compared to We ≅ 50 or We ≅ 185. An average contact time of 11.1 ms was recorded across all four superhydrophobic surfaces and was largely independent of the surface microstructure. Under subcooled conditions with possible phase change, surface micro-/nanostructure affects droplet impact dynamics beyond static wetting consideration. Our findings show that microstructure depth and solid-liquid contact fraction significantly influence droplet rebound and/or adhesion on subcooled surfaces. Contact times increased significantly as the surface temperature was decreased and partial adhesion of the droplet was detected if the contact time exceeded ~ 20 ms. Higher relative humidity led to frost formation and hence to greater energy dissipation and droplet pinning during the receding phase, preventing a full rebound, which was independent of the surface morphology. Overall, shallow-featured surfaces exhibited superior water repellency at subcooled temperatures, attributed to their lower solid-liquid contact fraction.

**Supplementary Information:**

The online version contains supplementary material available at 10.1038/s41598-025-14634-4.

## Introduction

Interaction between a droplet and a solid surface in the form of droplet impact is very common in nature and in many industrial applications. Such an interaction can enable new technologies (e.g., precisely controlled inkjet printing^1,2^ but may cause issues in other applications, especially if the surface is subcooled below the freezing point of the liquid droplet (e.g., icing on aircraft components and wind turbines)^3^. Controlling the interaction between water droplets and subcooled surfaces is therefore crucial in various fields to ensure the safe and efficient operation of equipment, machinery, and infrastructure. In anti-icing technologies for aircraft, preventing droplet freezing upon impact is vital to maintain aerodynamic performance and thus flight safety^4^. Similarly, in the automotive industry, the impact and freezing of water droplets affect windshield visibility. In refrigeration and air conditioning systems (e.g., where heat pumps and evaporators are used, especially in cold and humid climates), controlling droplet behavior can enhance system efficiency by preventing frost formation, which otherwise compromises heat transfer efficiency^5^.

With the rapid advancement of novel droplet-based technologies and the requirement for controlling phenomena governed by droplet impacts, many studies have focused on understanding the droplet dynamics and the factors that influence them^6,7^. The behavior of droplets, including spreading, receding, rebounding, and splashing, depends heavily on the conditions of both the droplet (i.e., its velocity, diameter, viscosity, density, surface tension, etc.), and the surface (mainly its wettability, micro- and nanostructure, and temperature). The intricate interplay of these parameters affects the droplet impact dynamics and causes different outcomes; under some conditions, the droplet may fully rebound off the surface, while it may partially or entirely adhere to it under different conditions^7,8^.

To characterize the experimental conditions and arrive at universal and generalized conclusions, the main parameters that influence droplet impact, spreading, and rebound can be characterized through multiple dimensionless numbers. The velocity of the droplet before the impact (*u*_0_) is usually given through the Weber number (We = $\:{\rho\:}_{\text{L}}{D}_{0}{u}_{0}^{2}{\sigma\:}^{-1}$), which expresses the ratio of inertial forces to the surface tension forces and is calculated from the droplet diameter before the impact (*D*_0_), its surface tension (*σ*), and density (*ρ*_L_). A droplet will start breaking apart via fingering of the lamella at higher Weber numbers, while at lower Weber numbers, the droplet will mostly stay homogenous without the creation of satellite droplets.

Similarly, the ratio of inertial forces to viscous forces is given by the Reynolds number (Re = $\:{\rho\:}_{\text{L}}{D}_{0}{u}_{0}{\mu\:}_{\text{L}}^{-1}$), where dynamic viscosity *µ*_L_ is considered in the denominator. The combination of the Weber and the Reynolds number is also known as the Ohnesorge number (Oh = We^1/2^Re^−1^).

Various surface engineering strategies have been employed to influence the droplet-surface interaction during the droplet impact, including superhydrophobic surfaces (SHS) and slippery liquid-infused surfaces (SLIPS). Most research focuses on changing the wettability of the surface, often in combination with modifying its micro- and nanostructure. In essence, surface wettability refers to if and how well a liquid can wet a surface. On the surface side, wettability is chiefly influenced by the surface’s roughness and chemical composition and is typically quantified by the static contact angle of a liquid droplet on the surface. Dynamic properties, such as advancing and receding contact angles and contact angle hysteresis (the difference between these angles), also provide important information in the characterization of surface wettability, especially when dynamic processes (such as droplet impact on a surface) are involved. In the extreme case of static contact angles above 150° and a low contact angle hysteresis (less than 10°), the surface is termed superhydrophobic. Such surfaces tend to repel water droplets if their velocity (or Weber number) is not excessive, thus also preventing freezing.

To describe the spreading and the bouncing behavior of a droplet on a solid surface, several characteristic quantities must be defined. One of them is the spreading factor ($\:\beta\:$ = $\:D\left(t\right)/{D}_{0}$), which represents the ratio between the width of the droplet at a given moment *D*(*t*) after the impact and the initial diameter of the droplet at the moment of impact (*D*_0_). Typically, the maximum spreading factor *β*_max_ is of interest. The value of the spreading factor when the droplet motion ceases or the latter loses contact with the surface can be used to analyze the shape of the droplet, the extent of retraction and the existence of a droplet rebound. To determine the maximum spreading factor, a multitude of models has been presented thus far, with various levels of complexity^9^. The simplest models only account for the Weber and Reynolds numbers. More advanced models are often given implicitly and also account for the static (*θ*), advancing (*θ*_*A*_), or the Young contact angle (*θ*_*Y*_). Some of the most common models are summarized in Table 1.

**Table 1:** Summary of the common models for predicting the maximum spreading factor.

**Author(s)**	Formulation	Eq.
Jones^10^	$\:{\beta\:}_{max}=\sqrt{\frac{4}{3}{\text{R}\text{e}}^{0.25}}$	(1)
Scheller and Bousfield^11^	$\:{\beta\:}_{max}=0.61{\left(\frac{\text{W}\text{e}}{\text{O}\text{h}}\right)}^{0.166}$	(2)
Asai et al.^12^	$\:{\beta\:}_{max}=1+0.48{\text{W}\text{e}}^{0.5}\text{exp}\left(-1.48{We}^{0.22}{Re}^{-0.21}\right)$	(3)
Roisman^13^	$\:{\beta\:}_{max}=0.87{\text{R}\text{e}}^{0.2}-0.4{\text{R}\text{e}}^{0.4}{\text{W}\text{e}}^{-0.5}$	(4)
Chandra and Avedisian^14^	$\:\frac{3\text{W}\text{e}}{2\text{R}\text{e}}{\beta\:}_{max}^{4}+\left[1-\text{cos}\left(\theta\:\right)\right]{\beta\:}_{max}^{2}-\left(\frac{\text{W}\text{e}}{3}+4\right)=0$	(5)
Mao et al.^15^	$\:\left[\frac{1}{4}\left[1-\text{cos}\left(\theta\:\right)\right]+0.2\frac{{\text{W}\text{e}}^{0.83}}{{\text{R}\text{e}}^{0.33}}\right]{\beta\:}_{max}^{3}-\left(\frac{\text{W}\text{e}}{12}+1\right){\beta\:}_{max}+\frac{2}{3}=0$	(6)
Ukiwe and Kwok^16^	$\:\left(\text{W}\text{e}+12\right){\beta\:}_{max}=8+{\beta\:}_{max}^{3}\left[3\left[1-\text{cos}\left({\theta\:}_{Y}\right)\right]+4\frac{\text{W}\text{e}}{\sqrt{\text{R}\text{e}}}\right]$	(7)
Aksoy et al.^17^	$\:3.18\frac{{\text{W}\text{e}}^{0.72}}{{\text{R}\text{e}}^{0.86}}{\beta\:}_{max}^{6.5}=\left(\text{W}\text{e}+12\right){\beta\:}_{max}-{\beta\:}_{max}^{3}\left[3\left[1-\text{cos}\left({\theta\:}_{Y}\right)\right]\right]-8$	(8)
Pasandideh-Fard et al.^18^	$\:{\beta\:}_{max}=\sqrt{\frac{We+12}{3\left[1-\text{cos}\left({\theta\:}_{A}\right)\right]+4\left(\frac{\text{W}\text{e}}{\sqrt{\text{R}\text{e}}}\right)}}$	(9)

In general, several outcomes of droplet impact onto a solid surface are possible. The droplet may be deposited onto the surface, it may splash, break up, or bounce (fully or partially). When a droplet rebound occurs, the restitution coefficient *ε* may be determined^9,19^defined as the ratio of droplet velocity after leaving the surface and before the impact. Finally, the contact time represents the time the droplet spent in contact with the surface during the impact and may critically influence the onset of nucleation if this impact occurs on a subcooled surface. The contact time (*τ*) generally scales with the liquid density *ρ*_L_, *D*_0_, and the surface tension of the droplet *σ* following the proportionality $\:\tau\:\propto\:{\left({\rho\:}_{\text{L}}{D}_{0}^{3}{\sigma\:}^{-1}\right)}^{1/2}$^20–22^. More specifically, the expression $\:\tau\:$ = $\:2.6$($\:{\rho\:}_{\text{L}}{D}_{0}^{3}{\left(8\sigma\:\right)}^{-1}$)^1/2^ can be used to predict the droplet contact time^23^.

Surface temperature is also known to significantly affect the outcome of droplet impact onto a solid surface^24–27^. If the surface is at an elevated temperature compared to the droplet’s temperature, heat and mass transfer phenomena may occur. When the surface is sufficiently cooled below the freezing point of the liquid, droplets may nucleate and freeze on the surface. During the contact with the subcooled surface, the droplet will first cool below the equilibrium freezing temperature in the supercooling stage. This will be followed by nucleation and recalescence, giving rise to the formation of a solid phase within the droplet during solidification. When the freezing is complete, the droplet will again cool below the freezing temperature towards the surface temperature.

Depending on the Weber number, surface temperature, and surface properties, the droplet may freeze during the impact or may instead fully rebound, which is preferential in anti-icing applications and a part of the focus of this manuscript^26^. For example, Guo et al.^28^ fabricated a post-array and a flat superhydrophobic surface to study the droplet impact dynamics, focusing on the contact time and the bouncing behavior, including the dependence on the surface temperature, Weber number, and surface frost were systematically investigated. Over a specific Weber number range, the pancake rebound regime with significantly shorter contact time appeared, which is beneficial to the anti-icing performance. Further analysis indicated that decreasing surface temperatures caused frost formation between the posts, thus reducing the capillary energy stored during the downward penetration and resulting in the failure of the pancake bouncing. This caused the droplets to adhere to the frosted surface at a sufficiently low temperature (especially at higher We) due to the mutual influence of nucleation and wetting transition. Alizadeh et al.^24^ investigated the impact dynamics of water droplets at We = 138 on surfaces ranging from hydrophilic to superhydrophobic and across a wide temperature range from below freezing (−15 °C) to near boiling conditions (85 °C). They found a very strong temperature dependence of the droplet retraction on hydrophilic surfaces but practically negligible effects on the superhydrophobic surface, where low viscous dissipation and low adhesion losses contribute to full retraction and droplet rebound. Keshavarzi et al.^27^ studied the impact dynamics of water droplets on hydrophobic-to-superhydrophobic silicone rubber surfaces at −20, −10, and 25 °C with a focus on droplet behavior in the form of deposition, bouncing, or splashing. Only a minor effect of temperature on droplet dynamics on microstructured surfaces was observed for a wide range of We and Re, and full bouncing only occurred on superhydrophobic surfaces with a static contact angle (CA) > 160 ° and a contact angle hysteresis (CAH) < 2° at temperatures above 0 °C for We < 110 and Re < 5000.

However, existing studies mostly focused on the effects of surface structure at room temperature with no investigation into the effect of subcooled substrates, with a few notable exceptions. Gao et al.^29^ studied droplet impacts on cold superhydrophobic surfaces, focusing on how surface supercooling (i.e., the temperature difference between the equilibrium freezing temperature and the surface temperature) and Weber number influence the impact dynamics. They found that surface supercooling does not influence spreading time, while it strongly influences late retraction time as it can lead to a transition between wetting regimes. Zhang et al.^30^ investigated impacting-freezing dynamics of a room temperature and supercooled water droplet on a cold surface. They found that the existence of a full rebound largely depends on the temperature of the superhydrophobic surfaces, while negligible effects of the Weber number (varied between 10 and 90) were observed. Naveen et al.^25^ studied the effect of droplet temperature and surface wettability on the droplet impact. They found that the spreading process is very comparable on differently wettable surfaces at low droplet temperatures, marking a similar conclusion to other studies where the surface temperature was varied instead. Zhang et al.^26^ investigated the impact of supercooled water droplets on cold superhydrophobic surfaces while varying the surface structure (single-tier nanoscale roughness, hierarchical roughness with regular posts, hierarchical roughness with random structures). A counterintuitive trend was observed with the hierarchical surface with random roughness performing the best at water-repellency at low droplet speed (1.4 m s^−1^), which the authors attribute to a shortened contact time compared to other surfaces. In contrast, at the high impact speed of 2.4 m s^−1^, the single-tier nanorough surface exhibited the best icephobic behavior, attributed to a lower liquid–solid contact area.

To influence the droplet-surface interaction and possibly promote droplet rebound and hence prevent freezing, several surface functionalization approaches have been presented, with imparting surface superhydrophobicity representing the most common surface modification goal. Various methods have been developed to achieve a superhydrophobic behavior of surfaces, including sol-gel coatings^31,32^etching^33^galvanic deposition^34^lithography^35^electrospinning^36^and layer-by-layer assembly^37^. As a further surface microengineering approach, laser texturing offers several advantages over similar methods due to its relatively low cost, applicability to a broad range of materials (including hard-to-machine materials^38^, and high throughput. Several existing studies^39–42^ combined laser texturing with surface hydrophobization to produce superhydrophobic surfaces capable of repelling impacting water droplets, either at room temperatures or at subcooled surface temperatures. However, most studies failed to systematically explore the influence of laser-processing parameters and patterns on droplet rebound and freezing prevention. Furthermore, several studies^43–48^ already compared models for predicting the maximum spreading factor of a droplet, but they primarily focused on varying the fluid properties and/or surface wettability. Additionally, several previous studies focused on the effect of surface microstructure on droplet-surface impact dynamics^49–51^. However, these studies used surfaces with well-defined surface features (e.g., grooves of various heights, widths and spacings). On the other hand, laser-textured surfaces are hierarchical in nature with a combination of microscale and nanoscale elements, making it hard to identify a single quantitative metric to comprehensively describe the surface topography for the purpose of modelling phenomenological processes. Our study aims to also evaluate the prediction accuracy specifically for structured superhydrophobic surfaces with varying morphologies, a comparison that is largely unexplored.

To comprehensively investigate how surface microstructure influences the spreading, rebounding, and freezing behavior of impacting droplets under varying surface temperatures and droplet velocities, several types of superhydrophobic surfaces were fabricated and analyzed in this study. This multi-parameter study integrates surface morphology, impact velocity, substrate temperature, and ambient humidity into a unified experimental framework, enabling an insight into how microstructure governs both dynamic and thermodynamic aspects of droplet behavior on engineered superhydrophobic surfaces. Specifically, the effects of structure depth (~ 3 μm vs. ~30 μm) and structure type (randomized textures vs. directional microchannels) were examined by preparing laser-textured aluminum surfaces with either stochastic or deterministic features. These surfaces were rendered superhydrophobic through a combination of nanosecond laser surface texturing and subsequent hydrophobization with a self-assembled monolayer. Their morphological and wetting properties were characterized using profilometry, SEM (*Scanning Electron Microcope*) imaging, and both static and dynamic contact angle measurements. Droplet impact experiments were then conducted using ~ 2.6 mm diameter water droplets at room temperature, impacting the surfaces from three different heights corresponding to Weber numbers of approximately 50, 120, and 185. Surface temperature was systematically varied from 25 °C to − 30 °C. High-speed videography was employed to capture droplet spreading dynamics, and the maximum spreading factor was compared with predictions from eight literature-based models. Unlike most previous studies, we evaluate the predictive accuracy of these models on real, laser-textured superhydrophobic surfaces with complex hierarchical morphologies, which have not been widely tested in this context. In addition to tracking impact outcomes—classified as full rebound, partial rebound, or adhesion—the effect of environmental humidity on droplet behavior and potential surface frosting was also studied for two representative superhydrophobic surfaces. Firstly, the surface preparation and characterization are described, followed by the experimental methodology for droplet impact testing. The results are then presented and discussed with a focus on how surface morphology affects spreading, rebound, and freezing behavior, ultimately providing insights into the mechanisms underlying the observed phenomena.

## Materials and methods

### Surface preparation and analysis

**Fig. 1:**
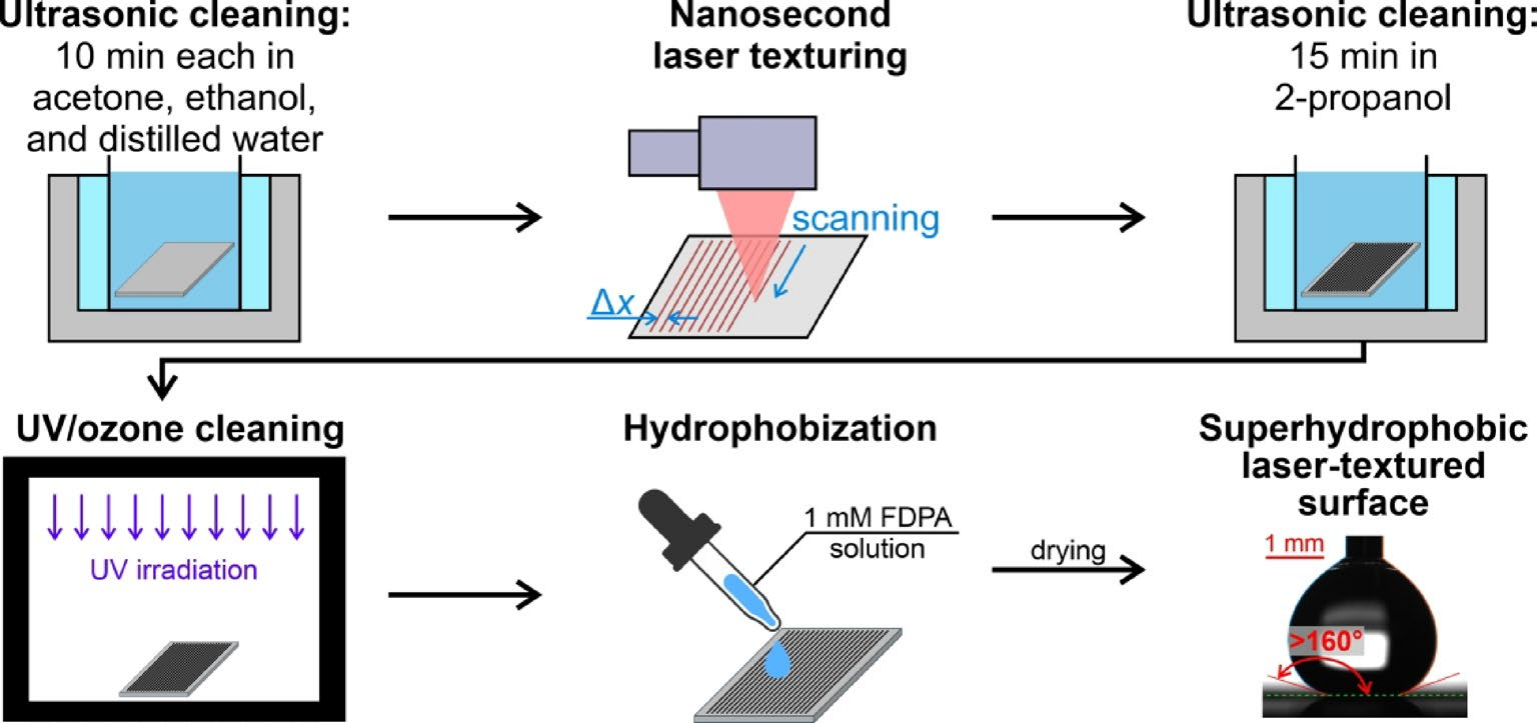
Surface functionalization procedure. Image created in CorelDRAW^®^ 2019.

Superhydrophobic laser-textured surfaces were fabricated on aluminum plates (1050 A H24, > 99.5% Al) with dimensions of 38 mm × 38 mm and a thickness of 1 mm. The entire functionalization process is summarized in Fig. 1.

A total of six samples were prepared and tested within this study. Firstly, an untreated reference sample “R” was prepared using only the first step of the functionalization procedure (i.e., three-stage ultrasonic cleaning). Secondly, a hydrophobized reference sample without the laser treatment “R-H” was prepared using all the steps outlined in Fig. 1 except for laser texturing. Lastly, four laser-textured and hydrophobized samples were prepared and are denoted as samples with a shallow texture (“ST*n*”) or a deep texture (“DT*n*”). The number “*n*” indicates the spacing between the two consecutive passes of the laser beam across the surface (either 25–50 μm). The four specific types of desirable surface features (deep/shallow, random/deterministic) were chosen in advance and the laser-texturing parameters to achieve them were experimentally determined. Specifically, the laser-texturing parameters were developed and fine-tuned through iterative development by fabricating a multitude of surfaces using various combinations of texturing parameters and selecting the most appropriate ones in each generation before performing cyclical optimization.

Before the laser treatment, the plates were sequentially cleaned in an ultrasonic cleaner for ten minutes in acetone, ethanol, and finally in distilled water. The laser texturing of the surfaces was performed utilizing a nanosecond fiber laser (JPT Opto-electronics Co., Ltd., M7 30 W MOPA; λ = 1064 nm). An F-Theta lens with a 70 × 70 mm² working area and a focal distance of 100 mm was used to focus the beam. The focused beam featured an approximate diameter of 25 μm on the surface during processing, with a manufacturer-reported laser beam quality of M^2^ < 1.3, and a maximum average laser source power of 30 W. The surfaces were prepared using a scanning pattern of equidistant parallel lines. The parameters employed for the laser texturing of the tested surfaces are summarized in Table 2. The average pulse fluence was calculated by taking into account the average laser power (*P*), the pulse frequency (*f*) and the focused beam diameter in the focal point (*D*_b_) to determine the average pulse fluence as *F* = 4*P*/(π*D*_b_^2^*f*).

**Table 2:** Laser-texturing parameters used to fabricate the functionalized surfaces.

Sample name	Line pitch [µm]	Scanning velocity [mm s^−1^]	Pulse frequency [kHz]	Pulse width [ns]	Average power [W]	Average fluence [J cm^−2^]
ST25	25	1650	110	45	18	28.8
ST50	50	1650	110	45	18	
DT25	25	400	80	100	18	39.6
DT50	50	400	80	100	18	

After laser texturing, the surfaces underwent an additional cleaning process in isopropanol using an ultrasonic cleaner for 15 min. Before carrying out hydrophobization, the samples were exposed to 15 min of UV/ozone cleaning using an Ossila UV/ozone cleaner to remove adsorbed species such as volatile organic compounds (VOC). The samples were hydrophobized using a solution of 3,3,4,4,5,5,6,6,7,7,8,8,9,9,10,10,11,11,12,12,12-henicosafluorododecylphosphonic acid (abbreviated to FDPA, CAS Nr. 252237-39-1, abcr GmbH). FDPA was dissolved in 2-propanol (Honeywell, ACS reagent, ≥ 99.5%) at a concentration of 1 mmol. A small amount of FDPA solution was pipetted onto each surface, forming a thin liquid layer that covered the entire surface. Subsequently, the surfaces were left to dry at room conditions for 15 min, followed by final drying in a preheated oven at 120 °C for another 60 min to remove the solvent and strengthen the bond between the FDPA and the substrate.

The morphology and chemical composition of the tested surfaces were analyzed using a scanning electron microscope (JEOL JCM-7000 NeoScope). Since aluminum is a good conductor of electricity, no additional sample preparation (other than cleaning with compressed air) was performed. SEM imaging was conducted using a secondary electron detector (SED) operated at an acceleration voltage of 5.0 kV under high vacuum. A working distance of ~ 12.5 mm was used together with the standard probe current. Images were taken at nominal magnifications of 300×, 800× and 2000×. Manufacturer-supplied NeoScope software environment was used for acquisition of images with a resolution of 2560 × 1920 pixels. The wetting properties of the samples were quantified using an Ossila goniometer for static contact angle measurements and a custom-designed experimental setup for dynamic contact angle determination. The roughness and surface profile of the samples were evaluated with a digital microscope (Keyence VHX-6000).

### Surface morphology and wettability

**Fig. 2:**
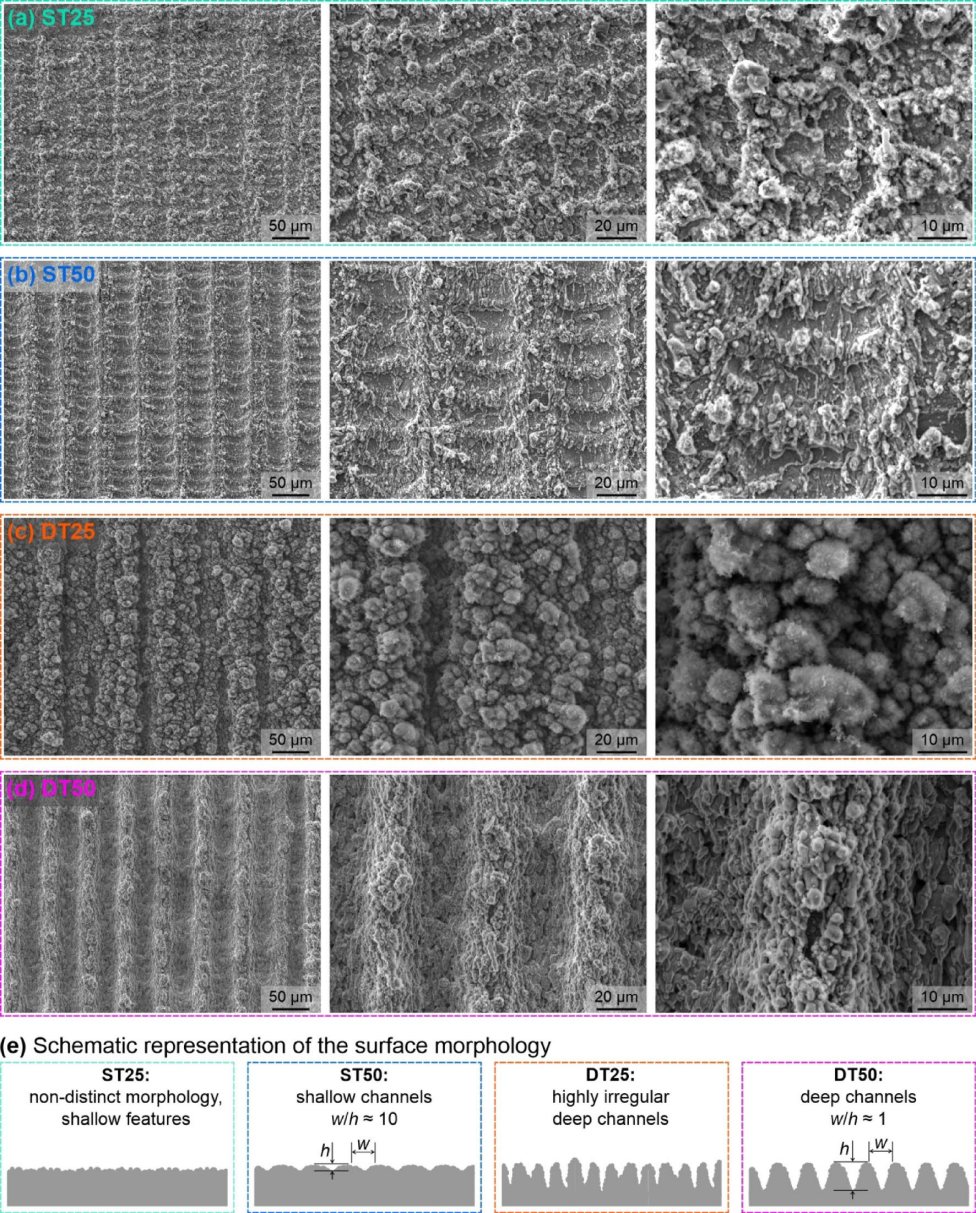
SEM images of the four laser-textured surfaces used in the study at three selected magnifications: (**a**) ST25, (**b**) ST50, (**c**) DT25, and (**d**) DT50; (**e**) schematic representation of the surface morphology of each test surface.

Figure 2 shows a comparison of SEM images taken on the four laser-textured surfaces. The ST25 surface treated with the lower laser pulse fluence and a 25 μm laser scanning line spacing exhibits non-distinct surface morphology where the laser-made channels are not well defined due to the lateral overlap of the subsequent irradiations of the surface. On the ST50 surface, which was also treated using the same laser-texturing parameters but with a doubled spacing of 50 μm, shallow channels are clearly defined. The channels are approximately as wide as the laser beam spot diameter on the surface (~ 25 μm).

Profilometric analysis revealed that the average depth of the channels is approx. 3.1 μm as reported in Table 3. On the other hand, much rougher microstructures are obtained using higher laser pulse fluence applied to both “DT” samples. On the DT25 surface with a 25 μm laser scanning line spacing, the channels are discernible but highly irregular, making quantification of their average properties difficult. On the DT50 surface with well-separated channels, however, the peak-and-valley microstructure is clean and an average channel depth of 26.8 μm was measured. SEM images of the untreated reference surface are provided in Figure S1 in the *Supporting Information* (section S1: *SEM images of the reference surface*).

Advancing and receding contact angles were measured on the superhydrophobic laser-textured surfaces to evaluate the droplet’s spreading and contracting behavior on the surface and are provided in Table 3. To determine the dynamic contact angles, droplet inflation/deflation method was used by utilizing a syringe pump. Advancing and receding contact angles were not measured on the two reference surfaces, and static contact angles were recorded instead. The untreated reference surface (R) exhibited a static contact angle of 63.4° ± 0.4 °, and its hydrophobized variant (R-H) exhibited an angle of 117.8° ± 0.4°. The roughness of the surfaces is given as the Sa (arithmetical mean height) roughness in Table 3 for the textured surfaces. For the untextured surfaces, the Sa roughness value was determined to be ~ 0.6 μm. Since the hydrophobized reference surface is covered only by a nanometric self-assembled monolayer of the FDPA, it is morphologically identical to the untreated surface. Additional roughness metrics are given in Table S1 in the *Supporting Information* (section S2: *Roughness metrics of the test surfaces*), while 3D topographical images are shown in Figure S2 in the *Supporting Information*. At 25 μm laser scanning line separation, the path of the laser beam across the surface partially overlaps with the previous pass, causing the existing channels to be partly filled-in and the overall morphology being non-distinct channels. At 50 μm separation, the laser-made channels are well-defined and high surface roughness can be observed on both the ST50 and DT50 surface compared to their counterparts with 25 μm spacing.

**Table 3:** Summary of the roughness and wettability of the functionalized surfaces.

Sample	$\:{\varvec{\theta\:}}_{\varvec{a}}$ (°)	$\:{\varvec{\theta\:}}_{\varvec{r}}$ (°)	CAH (°)	Sa (µm)	Surface features
ST25	167.0 ± 0.9	165.6 ± 1.0	1.4 ± 1.3	1.1 ± 0.2	Non-distinct shallow morphology
ST50	167.2 ± 1.3	163.9 ± 2.5	1.7 ± 2.8	2.4 ± 0.2	Shallow channels approx. *h* = 3.1 μm deep and *w* = 23.2 μm wide
DT25	163.3 ± 0.9	161.8 ± 1.3	1.5 ± 1.5	6.0 ± 0.5	Irregular deep channels
DT50	165.2 ± 1.1	163.3 ± 1.5	1.9 ± 1.9	9.2 ± 0.6	Deep channels approx. *h* = 26.8 μm deep and *w* = 26.8 μm wide

### Experimental setup for the evaluation of droplet impacts on subcooled surfaces

**Fig. 3:**
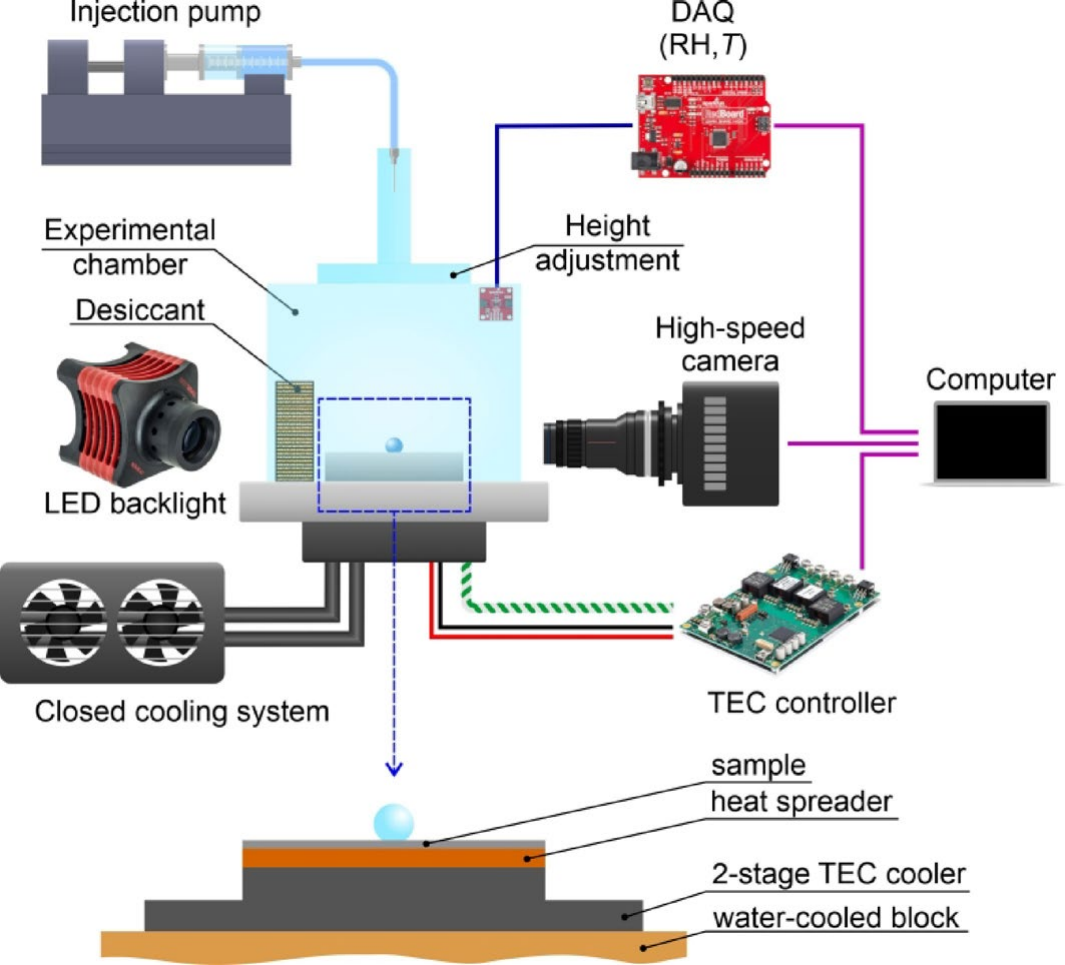
Schematic of the custom-made experimental setup for the evaluation of droplet impacts on subcooled functionalized surfaces. Image created in CorelDRAW^®^ 2019.

The evaluation of droplet-surface interactions was conducted using a custom-made experimental setup shown in Fig. 3. The experimental setup consisted of an acrylic glass chamber containing a cooling stage and auxiliary equipment. Cooling of the sample to the desired temperature was realized with a 2-stage Peltier element and an intermediate copper heat spreader. The hot side of the Peltier element was cooled by a water-cooling system. Temperature control was implemented with a Meerstetter TEC-1123-HV PID controller. A type K thermocouple was used to measure the temperature of the sample. Relative humidity between 4% and 7% was maintained inside the chamber during the measurements using molecular sieves to avoid frost accumulation on the test surface which would alter the surface-droplet interaction. The humidity and temperature were recorded using a SparkFun SHTC3 (Qwiic) sensor and a SparkFun RedBoard Qwiic Arduino controller. The typical accuracy of the relative humidity measurements is ± 2%RH (resolution: 0.01%RH), and the typical accuracy of temperature measurements is ± 0.2 °C (resolution: 0.01 °C). Two sets of experiments were repeated using silica gel as the desiccant, which provided a higher and more realistic relative humidity of 15–20%. For both humidity ranges, the ambient temperature in the experimental chamber was approx. 23 °C. To produce a droplet and change its impact velocity, a syringe pump, and a G27-size needle positioned at different heights above the test surface were used. The system produced droplets with a diameter of ~ 2.48 ± 0.09 mm. All calculations, such as that of the Weber number and the maximum spreading factor, are performed using the measured droplet diameter for each experiment. The diameter is measured from the high-speed video footage immediately before the droplet makes contact with the surface. The needle was mounted onto different 3D-printed holders designed to provide an appropriate height of the needle above the surface to achieve the necessary impact velocity for the desired Weber number values of 50, 125, and 200. The droplet temperature was the same as ambient temperature (23 ± 1 °C) as both the syringe, tubing and needle were exposed to the same ambient air as the entire experimental chamber. The water droplet impact on the surfaces was recorded using a Photron FASTCAM Mini UX100 high-speed video camera using a Laowa 100 mm f/2.8 2x Ultra Macro APO lens with a white LED backlight (Thorlabs SOLIS-2 C powered by a DC2200 controller). The droplet-surface interaction was imaged with droplet spreading parallel to the laser-made channels on all samples. Comparison of the maximum spreading factor values imaged parallel and perpendicular to the channels is included in the *Supporting Information* (section S3: *Maximum spreading factor versus the observation direction relative to the laser-made channels*), where it is shown that statistically significant differences between the two observation directions only arise on the two samples with deep textures (i.e., DT25 and DT50). Since all observations were made with the same orientation of the high-speed camera relative to the direction of the channels, no further investigation into this was made.

Experiments were conducted at reduced humidity levels to prevent frosting on the subcooled test surface, which could obscure the observation of fundamental droplet-surface interactions and introduce an additional, difficult-to-control variable as the rate of frost accumulation is not trivial to control. However, in real-world applications, surfaces are often exposed to higher humidity levels, which could influence both droplet dynamics and surface ice formation. A potential implication of increased humidity is the presence of pre-existing condensation on the surface, which could alter the wetting state and impact the droplet’s spreading and rebound behavior. Additionally, higher humidity levels promote faster frost formation, potentially modifying surface properties over time. While this was not investigated in the present study, future research works might explore how such humidity-induced changes affect droplet impact dynamics, providing a more comprehensive understanding of real-world performance.

### Measurement protocol

The droplet impact measurements on all surfaces were conducted at atmospheric pressure and in a room-temperature atmosphere with reduced relative humidity. Prior to conducting the droplet impact measurements, the desired relative humidity was achieved by passively dehumidifying the experimental chamber with desiccant for several minutes until the relative humidity value stabilized. 8 µL (± 0.9 µL) droplets of double distilled water were produced using a syringe pump operated at a flow rate of 150 µL min^−1^. The droplets were released onto the surface which was kept at the desired temperature with a PID controller. Three different heights were used to obtain the desired Weber number values. Statistical analysis of droplet diameter and impact velocity showed that the following average Weber numbers and their standard deviations were obtained: 50 ± 2 for the first height, 120 ± 6 for the second height, and 185 ± 8 for the third height. These average values will later be reported when presenting the results of droplet impacts as a function of the Weber number. The high-speed camera was used to record the impact process at 5000 fps. The footage was then post-processed to extract the impact velocity, the spreading factor, and other relevant parameters. The impact velocity was calculated by tracking the movement of the droplet over time immediately before its first contact with the surface. The post-processing of the recorded data was performed using custom-developed algorithms in the MathWorks MATLAB environment. The measurements were repeated three times for each combination of the surface, the surface temperature, and the impact velocity. Automatic processing of the high-speed footage was employed where possible, while manual analysis was performed as needed at the highest Weber number measurements due to droplet splashing.

## Results and discussion

### Comparison of the measured maximum spreading factors with model predictions at room temperature

**Fig. 4:**
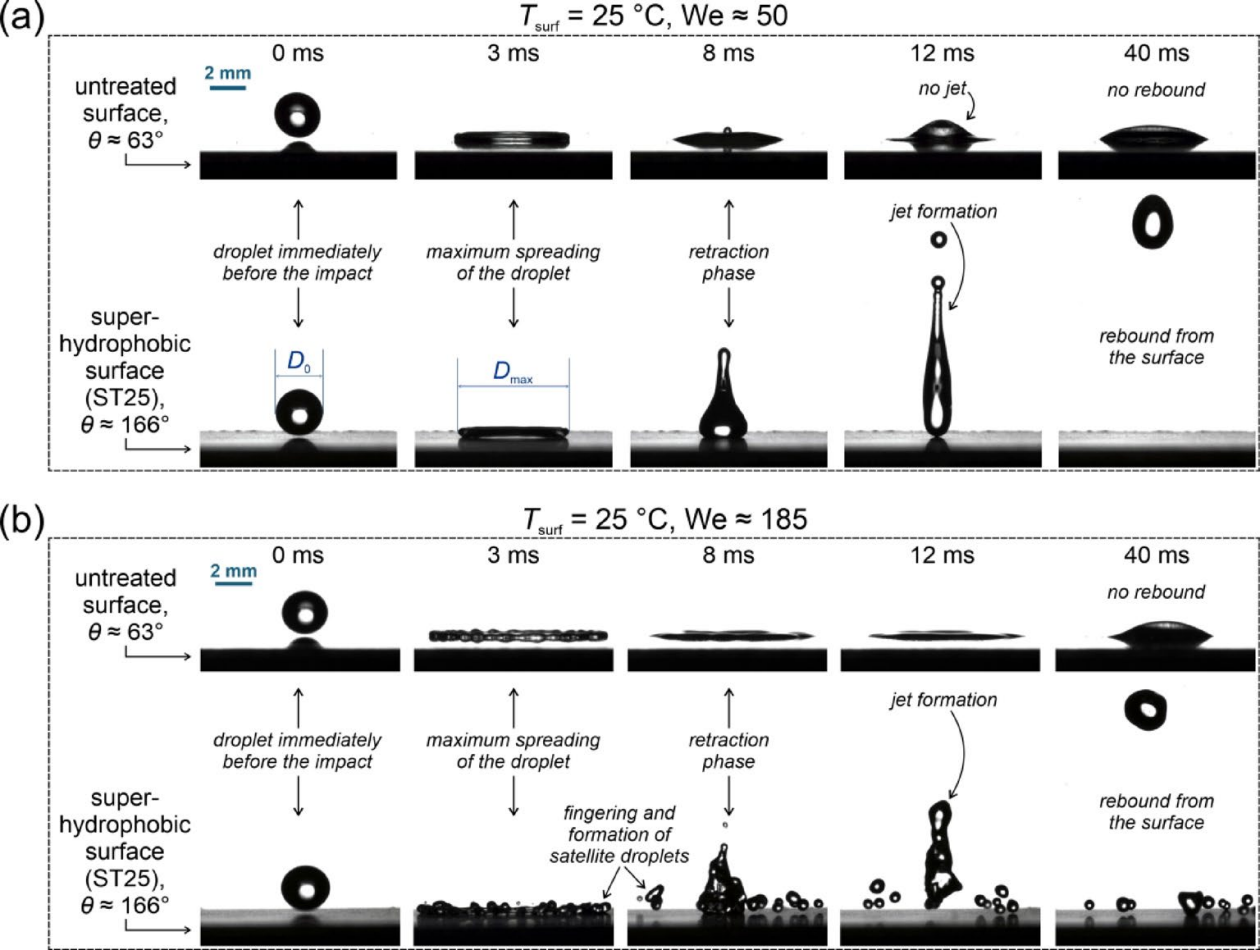
Impact dynamics of a 2.6 mm water droplet on two surfaces with different wettability for We ≅ 50 (**a**) and We ≅ 185 (**b**). All measurements were performed with the droplet and the surface at ambient temperature.

Figure 4 illustrates the effect of Weber number and the state of the surface on the spreading and retraction of the droplet and the existence of a rebound at the end of the spreading phase. In particular, it shows the impact dynamics in the form of high-speed camera snapshots for two surfaces (the untreated surface “R” and one of the superhydrophobic surfaces “ST25) and at two Weber numbers. At the lower Weber number of ~ 50 [Fig. 4(a)], the spreading phase is comparable on both surfaces, while significant differences occur during retraction, where the triple contact line is pinned on the slightly hydrophilic reference surface. On the other hand, the contact line moves freely on the superhydrophobic surface, resulting in the droplet fully contracting, forming a jet, and eventually losing contact with the surface during the rebound. At a higher Weber number [Fig. 4(b)], higher droplet inertia causes fingering on the edge of the droplet during the spreading phase on both surfaces. Fingering is more pronounced on the superhydrophobic surface, where secondary satellite droplets break off the flattened droplet around the time of maximum spreading (~ 3 ms after the initial impact). A pinned contact line is again present on the reference surface while full retraction, jetting, and eventual rebound of the main droplet are present on the superhydrophobic surface, alongside smaller satellite droplets, which remain on the surface.

**Fig. 5:**
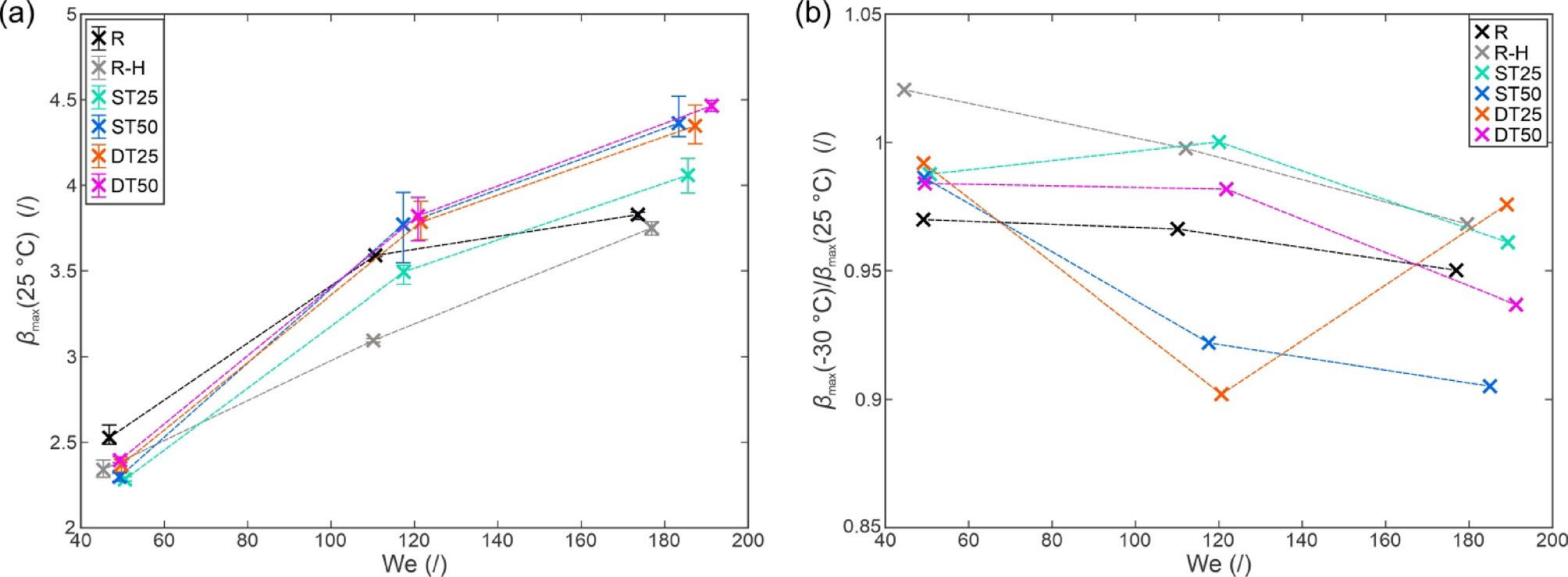
Maximum spreading factor at a surface temperature of 25 °C versus the average We (**a**) and the relative maximum spreading parameter at a surface temperature of – 30 °C (normalized with the value at 25 °C) versus the average We (**b**).

The initial and maximum droplet diameters were extracted from high-speed videos as shown in Fig. 4(a) and were used to calculate the maximum spreading factor. The values of the maximum spreading factor were analyzed for measurements with the surfaces at room temperature to determine the effect of the surface morphology on the droplet spreading. The results of these measurements at three average We values are shown in Fig. 5(a). A negligible impact of surface morphology was observed at We ≅ 50, and no conclusive trend was identified at We ≅ 120. However, 6–17% higher maximum spreading factor was observed on superhydrophobic surfaces compared with the untreated reference surface at We ≅ 185. This result points to decreased energy dissipation during droplet spreading due to the reduced solid-liquid contact on the superhydrophobic surfaces where the Cassie-Baxter heterogeneous wetting regime is present. Furthermore, the shape of the wetting front is different on superhydrophobic surfaces (resembling a hemisphere) and the rim of the lamella never touches the solid substrate^52^. However, all superhydrophobic surfaces exhibited similar maximum spreading factors, with only a slight deviation to ~ 7% lower values observed on the ST25 surface with shallow, non-distinct microstructure.

To investigate the effect of decreased surface temperature on the spreading of the droplet, the measured maximum spreading factors at −30 °C (i.e., the lowest temperature in this study) were normalized using the maximum spreading factor values at room temperature with the results shown in Fig. 5(b). At the lowest We, a reduction of the *β*_max_ in the region of 1–3% was observed for 5 out of 6 surfaces. Our observations match those reported by Yao et al.^53^who observed a 4.3% decrease in the maximum spread factor when decreasing the temperature of a superhydrophobic surface from 20 °C to −60 °C at We = 63. In all cases except for the DT25 surface, the ratio between *β*_max_ at −30 °C and 25 °C decreased further with increasing Weber number, albeit the final values at We ≅ 185 showed only a ~ 2.5–9.5% decrease compared to the measurements at room temperature. While the results confirmed that the reduced surface temperature affects the spreading phase during the droplet impact, which most likely arises due to increased viscosity and viscous dissipation due to locally decreased droplet temperature, the decrease is small even at −30 °C, and no clear effect of the surface microstructure could be observed. Additionally, the maximum spreading factors measured at all temperatures and all We are shown in Figs. S3-S5 in the *Supporting Information* (section S4: *Maximum spreading factor versus the surface temperature*).

A range of recent studies^54–58^ supports the conclusion that, under the conditions used in this study, droplet solidification does not affect maximum spreading during the spreading phase. Specifically, ice crystal growth velocities are much lower than the measured lamella velocities (e.g., 1.61–2.46 m/s at We ≅ 50–90)^59^meaning freezing cannot arrest spreading, even if nucleation occurs shortly after impact. Superhydrophobic surfaces, such as those used in this study, further reduce the likelihood of nucleation and heat transfer due to minimal solid–liquid contact. While other works have shown slight reductions in spreading on cold or hydrophilic substrates, these effects are minimal or negligible under the test conditions used in the present study. Therefore, the observed maximum spreading remains unaffected by surface subcooling or freezing during the spreading phase.

**Fig. 6:**
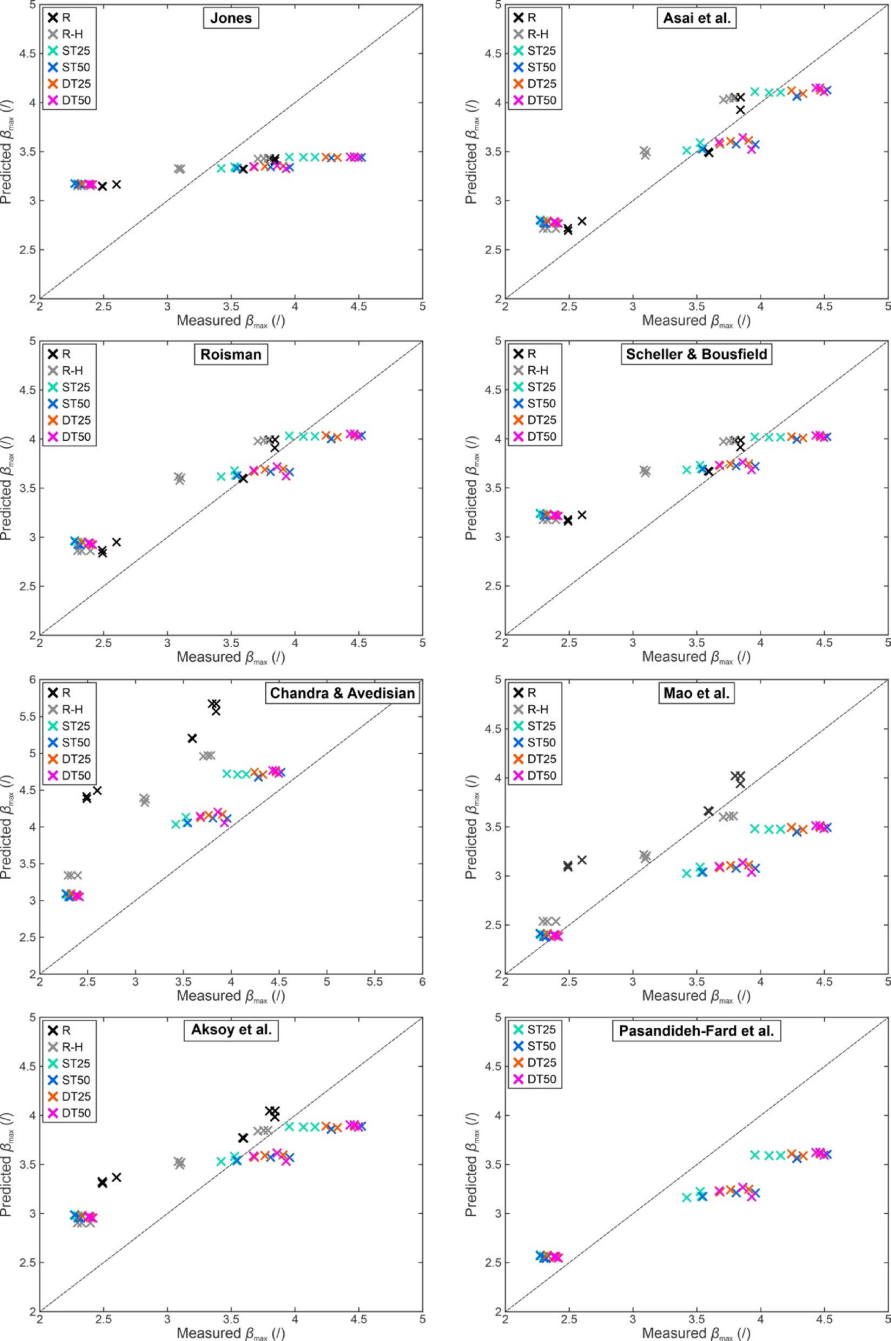
Measured vs. predicted maximum spreading factor at a surface temperature of 25 °C for eight models.

The investigation was continued by comparing the measured maximum spreading factors on surfaces at room temperature to predicted values from eight models in Table 1 as shown in Fig. 6. The models significantly differ in complexity and may be based solely on the Reynolds and Weber numbers or also include the static, Young’s or advancing contact angle. While none of the models can account for the specific surface microstructure, results in Fig. 5 indicate the relatively minor importance of the microstructure in spreading of the droplet (within the range tested in this study).

The results in Fig. 6 indicate that most models reasonably predict the maximum spreading factor, albeit with deviations of > 10%. On the other hand, two models (Jones’^10^ and Chandra and Avedisian’s^14^ provided a completely unsuitable fit, which, in the case of Jones’ model, should be attributed to the model’s simplicity as it only accounts for the Reynolds number. A statistical evaluation of the predictions is shown in Table S2 in the *Supporting Information* (section S5: *Statistical evaluation of different maximum spreading factor models*). Mean absolute percentage error (MAPE) is used to compare different models, and the lowest value of 9.5% was determined for the model by Asai et al.^12^followed by models by Mao et al.^15^ and Roisman^13^ with a MAPE of 11.4% and 11.8%, respectively.

Regardless of how well individual models fit the data collected in the present study, no trend in terms of the effect of surface morphology on the deviation of experimental observations from model predictions was observed. It should be noted that the models do not include parameters describing the surface micro- and nanostructures and, therefore, cannot capture the differences in the maximum spreading factor governed by surface morphology.

### Outcome of the droplet impact as a function of surface temperature and the Weber number

**Fig. 7:**
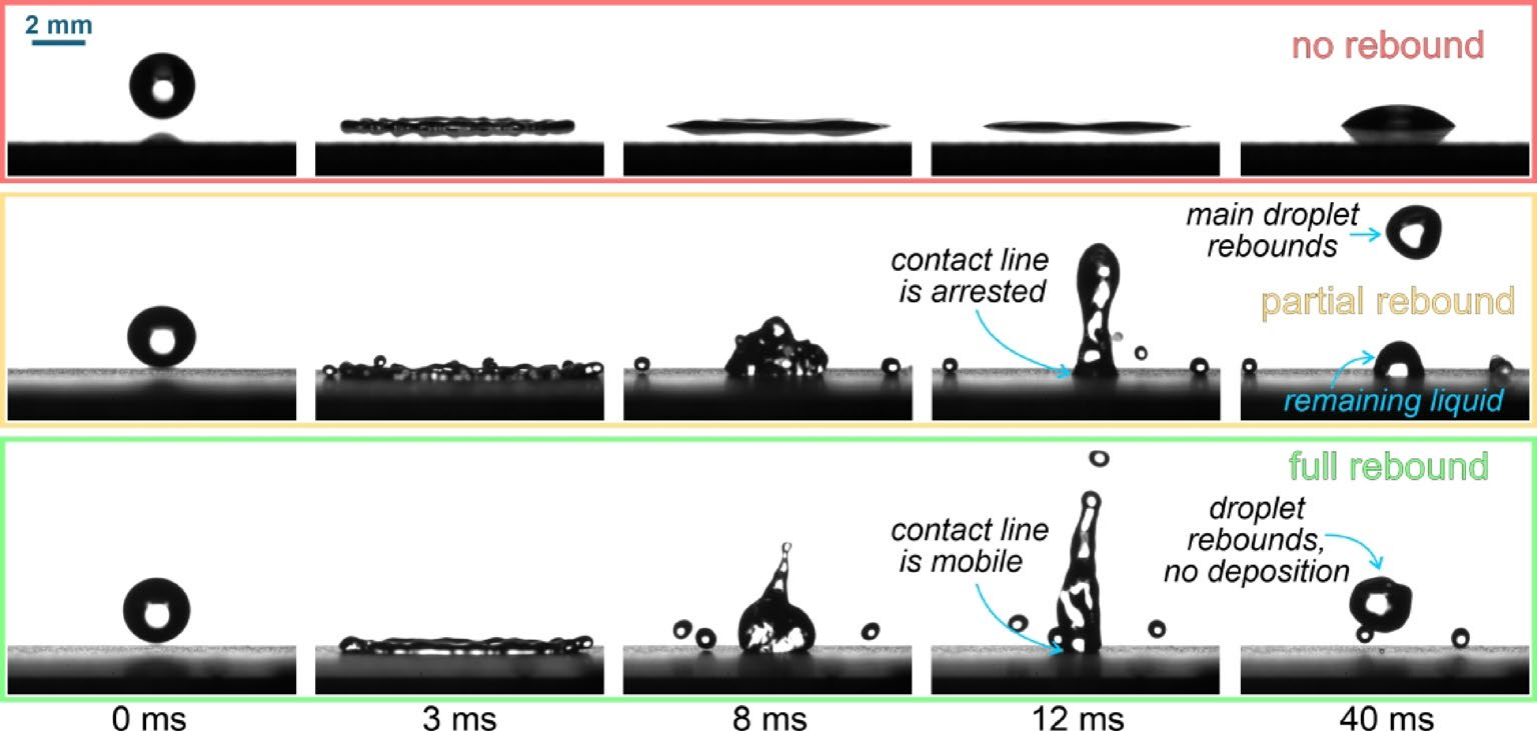
Possible outcomes of the droplet-surface interaction: no rebound with full adhesion (top), partial rebound with some leftover liquid on the surface at the location of the impact (middle), and full rebound of the droplet (bottom).

To continue the investigation of the effect of surface microstructure on the droplet’s interaction with engineered surfaces, droplet rebounds were analyzed on all six surfaces at different temperatures (25 °C, −15 °C, −20 °C, −25 °C, and − 30 °C) and at three average Weber numbers (50, 120, 185). The aim was to determine whether the droplets adhere to or bounce off the surface for the given combination of surface temperature and We. The outcomes of the droplet impact are shown in Fig. 7 and were classified into three categories: *(i)* green: full rebound with no liquid remaining on the surface (although mobile satellite droplets may remain on the surface), *(ii)* yellow: partial rebound with some leftover liquid, or *(iii)* red: no rebound with adhesion of the droplet and possibly its solidification. The possible outcomes are shown in Fig. 7.

**Fig. 8:**
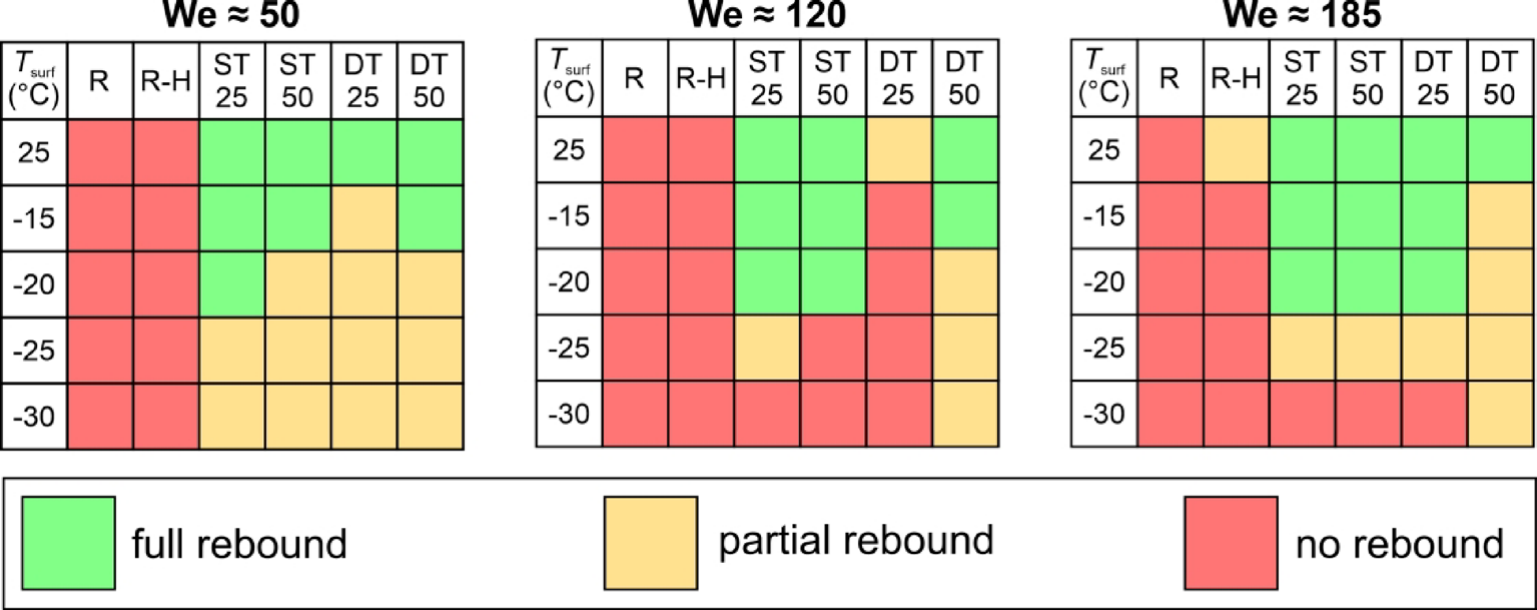
Droplet impact outcomes versus surface temperature for three average Weber numbers.

Three measurements were made for each combination of surface temperature and We, and the results of average droplet impact outcomes are shown in Fig. 8.

The results in Fig. 8 show that the slightly hydrophilic untreated surface “R” expectedly fails to rebound the impacting water droplet at all temperatures. The same applies to the hydrophobized reference surface “R-H”, which only partially rebounds droplets at room temperature and the highest Weber number. With a contact angle of ~ 118°, the R-H surface exhibits wetting in the Wenzel wetting regime and can thus not be expected to successfully repel water droplets under most circumstances. On the other hand, all four superhydrophobic surfaces achieved a rebound of the impacting droplet at all Weber numbers at room temperature, which is attributable to their superhydrophobic behavior. However, at subzero temperatures, droplet rebound was no longer guaranteed on some surfaces and significant differences arose.

The two surfaces with shallow textures (ST25 with non-distinct structures and ST50 with shallow channels) managed to fully rebound water droplets both at −15 °C and − 20 °C (except for ST50 at We ≅ 50 and − 20 °C). However, some partial rebounds or a complete lack of a rebound were detected at −25 °C, transitioning to droplet adhesion with no rebound at −30 °C for both surfaces and at all We except We ≅ 50, where a partial rebound was observed on both surfaces with shallow features. Finally, the DT50 surface with well-ordered deep microchannels managed to either entirely or at least partially rebound droplets at all temperatures and Weber numbers. Nevertheless, partial droplet adhesion was detected at all subzero tests at the highest Weber number and also at We ≅ 120 for − 20 °C or below.

The main barrier to liquid penetration into the surface microstructure and consequent impalement of an impacting droplet is the capillary pressure provided by the surface structures, which can be calculated based on the surface tension of the liquid *σ*, advancing contact angle *θ*_*A*_ and the width of the grooves *w* (note that *w* = 2*r*):

10 $$\:{p}_{C}=-\frac{4\sigma\:\text{cos}\left({\theta\:}_{A}\right)}{w}$$

It has been shown that the effective “water hammer” pressure of the droplet impact is likely the main cause of liquid penetration into the microstructure if its value exceeds the capillary pressure^60–62^:

11 $$\:{p}_{WH}=k\rho\:{c}_{s}{u}_{0}$$

In Eq. (11), *k* is a surface-property-dependent empirical constant with typical values on the order of 5∙10^−4^ – 2∙10^63^, *ρ* is the liquid density, *c*_*s*_ represents the speed of sound in the liquid and *u*_0_ is the droplet’s impact velocity. It is also possible for partial pinning and adhesion (i.e., partial rebound) to appear if the capillary pressure is lower than the water hammer pressure but higher than the dynamic pressure of the droplet:

12 $$\:{p}_{D}=\frac{\rho\:{u}_{0}^{2}}{2}$$

Following the example by Dash et al.^63^the critical velocity of droplet impalement due to a Cassie-to-Wenzel wetting transition was estimated using the upper and lower values of the constant *k* in Eq. (11) by solving for the velocity at which the capillary pressure equals the sum of the dynamics and the water hammer pressure. Considering the groove widths and advancing contact angles of the ST50 and DT50 surfaces given in Table 2, the following critical velocities were determined. At *k* = 2∙10^−3^ and *k* = 5∙10^−4^, the critical velocity on the ST50 surfaces lies between 2.77 m s^−1^ and 4.24 m s^−1^, respectively. On the DT50 surface, corresponding values of 2.46 m s^−1^ and 3.88 m s^−1^ were determined. Given the worst-case critical velocity of 2.46 m s^−1^, all velocities used within the study including the droplet velocity at We ≅ 185 (*u*_0_ ≅ 2.31 m s^−1^) are lower than the critical velocity needed to overcome the capillary pressure needed for water to penetrate into the laser-made grooves. Therefore, a complete droplet rebound with no temperature effects (i.e., with droplet and surface both at ~ 25 °C) is expected, which agrees well with the results presented in Fig. 8. However, the ability of most surfaces to completely rebound impacting droplets was better at We ≅ 185 than at We ≅ 120, which does not follow the expected trend of the monotonically decreasing rebound performance with increasing impact velocity (i.e., with increasing We) as the threshold critical velocity is approached.

Furthermore, the overall best performance (considering all temperatures and Weber numbers) in repelling impacting droplets was observed on the ST25 surface with shallow, non-distinct structures, also going against the expectation that microscale grooves (such as the laser-made open microchannels on ST50 and DT50 surfaces) would provide the most favorable droplet impact resistance. Considering the roughness scale of the ST25 surface of ~ 1.1 μm, the theoretical droplet velocity to ensure sufficient water hammer and dynamic pressure to overcome the associated capillary pressure would exceed 19.8 m s^−1^.

Deeper grooves should theoretically result in a higher critical impalement pressure and increase the robustness against liquid impalement to maintain the Cassie-Baxter wetting state^62,64^. However, this trend was not observed, which could be attributed to the hierarchical nature of the ST50 and DT50 surfaces, which are both covered with micro- and nanoscale features resulting from laser texturing, thus exhibiting superhydrophobicity both on the top of the peaks of the grooves and in the valleys in between the peaks, therefore resisting droplet impalement regardless of the channel depth.

**Fig. 9:**
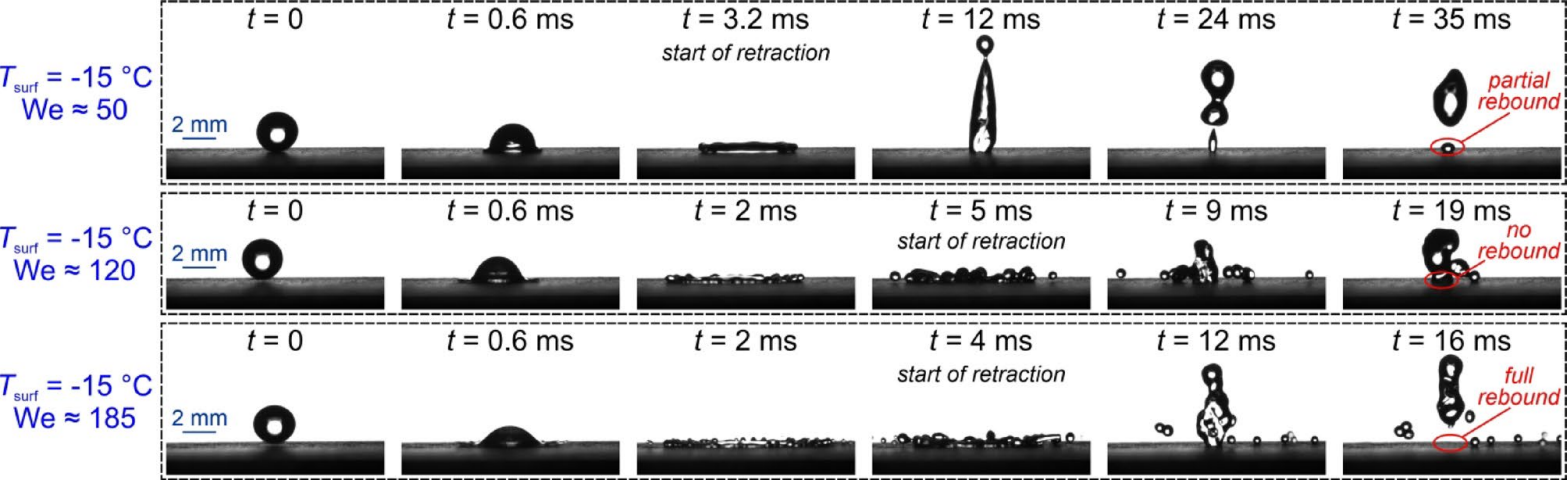
Different droplet impact outcomes on the DT25 surface at −15 °C for different average Weber numbers.

The most interesting are the results for the DT25 surface with highly irregular deep microstructure, where an intermediate regime of complete droplet adhesion was observed at We ≅ 120, while partial rebounds down to −25 °C were observed at We ≅ 50 and full rebounds down to −20 °C at We ≅ 185. At We ≅ 120, partial adhesion of the droplet was observed already at room temperature. At first glance, this is contrary to the initial expectations, which suggest that for this specific surface, the droplet pinning does not arise from the water hammer pressure (being positively correlated to the impact velocity), but rather from the pressure generated during the retraction stage of the rebound (i.e., the recoil pressure), as suggested by Lee et al.^65^. As such, one can assume that at We ≅ 50, the relatively low recoil pressure induces localized entrapment below the droplet’s center, resulting in a partial droplet rebound. Increasing the Weber number to approximately 120 leads to greater droplet spreading, thereby increasing recoil pressure, which promotes droplet pinning over a larger area. At this point the adhesion force overcomes inertia, preventing the droplet from rebounding from the surface. Finally, at We ≅ 185, the rebound transitions to splashing, resulting in large energy dissipation caused by the formation of smaller droplets. The latter process reduces the recoil pressure, causing the droplet to again fully rebound. A comparison of the droplet rebound on the DT25 surface at three Weber numbers is shown in Fig. 9. Due to the initiation of splashing behavior at We ≅ 120, the retraction phase starts about 5 ms after the droplet impact. In contrast, for the We ≅ 50 and We ≅ 185 cases, this timeframe is 20% and 36% shorter, respectively. A prolonged droplet/surface contact for the We ≅ 120 cases can therefore increase the possibility of freezing.

Further analysis can be found in the *Supporting Information* in sections S6-S8: *Snapshots of droplet impacts at various temperatures and We* ≅ *50*; *Snapshots of droplet impacts at various temperatures and We* ≅ *120*; and *Snapshots of droplet impacts at various temperatures and We* ≅ *185*, where snapshots of the droplet impact process are compared for various combination of surfaces, surface temperatures, and all three average Weber numbers.

**Fig. 10:**
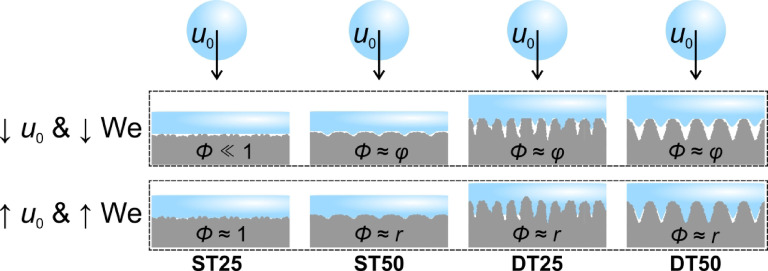
Schematic representation of liquid penetration and the solid-liquid contact area at a low droplet velocity (upper row) and a high droplet velocity (bottom row).

Overall, two surfaces can be singled out based on their droplet repellency and prevention of freezing. Firstly, the ST25 surface with shallow features and no discernible channels (Sa ≅ 1 μm) was the only surface to ensure a full rebound down to −20 °C at We ≅ 50 and also provided a partial rebound at all Weber numbers down to −25 °C. Due to the low roughness of the ST25 surface the solid-liquid contact area (*Φ*; 0 ≤ *Φ* ≤ *r*), representing the ratio between the actual solid-liquid contact area and the projected surface area, is very small at a low droplet impact velocity when the dynamic and water hammer pressures are low (i.e., not enough to force the liquid into the microstructure of the surface) as the liquid is locally supported by individual hydrophobized micro- and nanoparticles present on the surface (see Fig. 2). Compared to other surfaces with more pronounced morphology with characteristic dimensions on the scale of tens of micrometers, the actual solid-liquid fraction is likely lower than the solid fraction *φ* (0 ≤ *φ* ≤ 1) in the Cassie-Baxter wetting model, which is assumed to be present on other surfaces, where the droplet is supported by the local microstructure peaks. This is shown schematically in Fig. 10. At higher droplet velocities, the droplet protrudes deeper into the microstructure, making the liquid-solid contact area (*Φ*) close to or larger than the apparent contact area. Its value approaches unity on the ST25 surface due to the low roughness. Still, the contact area fraction is much higher on other surfaces with up to one order of magnitude higher microstructural features and approaches the roughness factor *r* of the individual surface, which is used in the Wenzel wetting model (*r* ≥ 1). A lower solid-liquid fraction results in a lower ice nucleation rate at the interface in accordance with the classical nucleation theory, decreasing the likelihood of nucleation and thus increasing the likelihood of a successful droplet rebound at low surface temperatures^26^. Furthermore, the Cassie-Baxter wetting regime is more easily maintained homogeneously across the surface when only submicron morphological features are present. According to Eq. (10), smaller characteristic dimensions significantly increase the capillary pressure and thus the resistance against the Cassie-to-Wenzel transition, which locally leads to “sticky” areas with a nano-Wenzel wetting regime^66^. Specifically, the capillary pressure exceeds 254 kPa on the ST25 surface when taking the Sa value as the characteristic size of the surface features.

On the other hand, the droplet repellency was not the highest on the DT50 surface with well-ordered deep microchannels, but it was the only surface without detected full adhesion even at −30 °C. Instead, this type of surface exhibited a partial rebound at most combinations of surface temperature and Weber number. While a full rebound is preferable to partial adhesion, the latter can be useful to reduce the rate of ice accumulation on the surface since only a part of the water droplet remains on the surface, thus making the DT50 surface the preferable choice at surface temperatures of −25 °C and − 30 °C. It appears that the well-ordered channels provide high capillary pressure, which is estimated to be ~ 10.4 kPa using Eq. (10). However, local pinning of the droplet appears to take place due to the vertical penetration of the droplet into the microstructure at the center of impact, adhering a part of the droplet to the surface and preventing a full rebound.

**Fig. 11:**
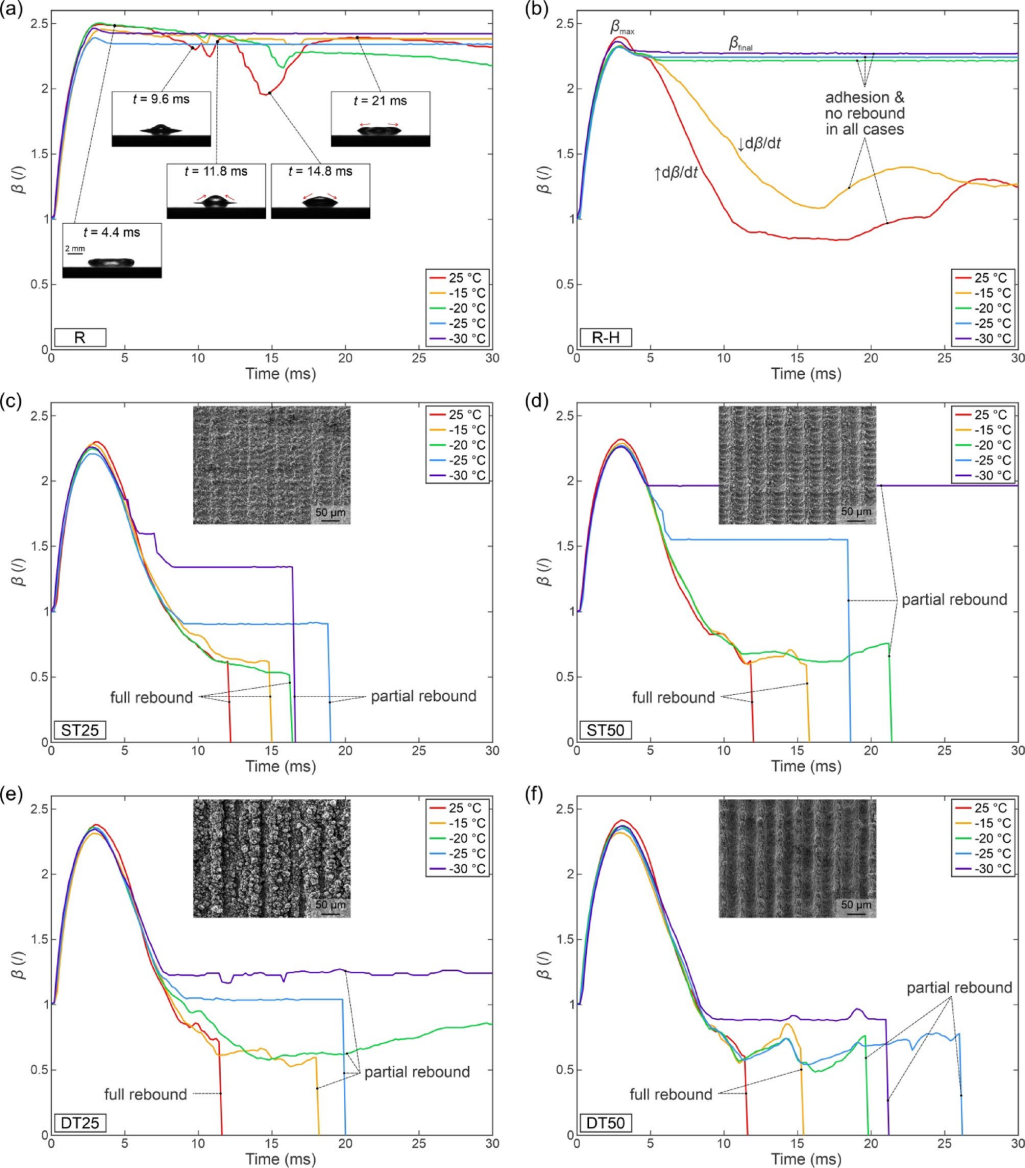
Spreading factor versus time for different types of surfaces: (**a**) untreated reference “R”, (**b**) hydrophobized reference “R-H”, and laser-textured surfaces with different morphologies (**c**-**f**) for We ≅ 50.

A further analysis of temperature-dependent and surface-dependent droplet rebound behavior is shown in Fig. 11, illustrating the temporal evolution of the spreading factor *β* at We ≅ 50 for one of the measurements performed on each surface at the given conditions. In all cases, the maximum diameter of the droplet was tracked from the moment of first contact with the surface to the moment of rebound (if it was present), hence, a sharp drop of *β* to zero is evident for full rebounds. Figure 11(a) shows that practically no retraction of the droplet is observed on the slightly hydrophilic reference surface “R” with the final spreading factor (after the droplet motion and oscillations cease) being roughly equal to its maximal value (*β*_final_/*β*_max_ ≅ 1). As evident in Fig. 5(a), only a small decrease in the maximum spreading factor compared to room temperature tests is observed even at the lowest temperature of −30 °C. Since the spreading phase is only ~ 3 ms long, there is insufficient time for the droplet to cool down enough for ice nucleation to take place. Hence, the triple contact line is not arrested, and the droplet reaches a spreading factor similar to what was observed during the room temperature tests. A notable retraction phase of the spreading is noticeable on the hydrophobic smooth reference surface “R-H” shown in Fig. 11(b), where the droplet contracts back to roughly its pre-impact diameter (*β*_final_ ≅ 1) at room temperature (see Fig. 5(b)). The same outcome is reached at a surface temperature of −15 °C, albeit with a slower retraction (less steep d*β*/d*t* slope).

On the other hand, the droplet’s final spreading factor stays close to its maximum value at −20 °C or below. From this, we estimate that approx. 5 ms is necessary for the droplet to cool down enough for the thinnest part of the lamella to start freezing, resulting in the pinning of its rim and an arrest of droplet motion since retraction is prevented (see Fig. 9). The necessary time for this phenomenon highly depends on the thermal effusivity of the substrate (with the aluminum boasting a high effusivity value thus being able to cool down the droplet more rapidly) and the surface temperature as shown by multiple investigations into the freezing of impacting droplets on substrates with different thermal properties^57,67,68^. The obtained values for the droplet motion arrest agree with those recorded on similar surfaces under similar experimental conditions, such as those shown by Gorin et al.^57^.

On the superhydrophobic surfaces shown in Fig. 11(c-f), the temporal evolution of the spreading factor tends to exhibit a sharp drop to zero, which indicates a droplet rebound, either full or partial. In all four cases, a full rebound in approx. 11–12 ms is observed at room temperature, but the contact time increases with decreasing surface temperature, which is analyzed in the next section. At low surface temperatures, the droplets begin to experience adhesion to the surface without a (full) rebound, resulting in a constant *β* at a value above zero.

The interplay between surface micro-/nanostructure and droplet impact dynamics extends beyond conventional static wetting considerations, particularly under subcooled conditions where solid-liquid phase change may take place. Our findings highlight that the microstructure depth and the solid-liquid contact fraction critically influence droplet rebound and adhesion. Shallower microstructures (3 μm depth) exhibit lower solid-liquid contact fractions, enhancing water repellency at subcooled temperatures compared to surfaces with deeper features (30 μm depth). These results underscore the importance of considering surface morphology—not just roughness magnitude—when evaluating superhydrophobic surfaces for anti-icing applications. While the surface morphology was shown not to have an impact on the maximum spreading factor at room temperature, the rebound behavior was vastly different and morphology dependent under subcooled conditions.

### Analysis of droplet contact time

**Fig. 12:**
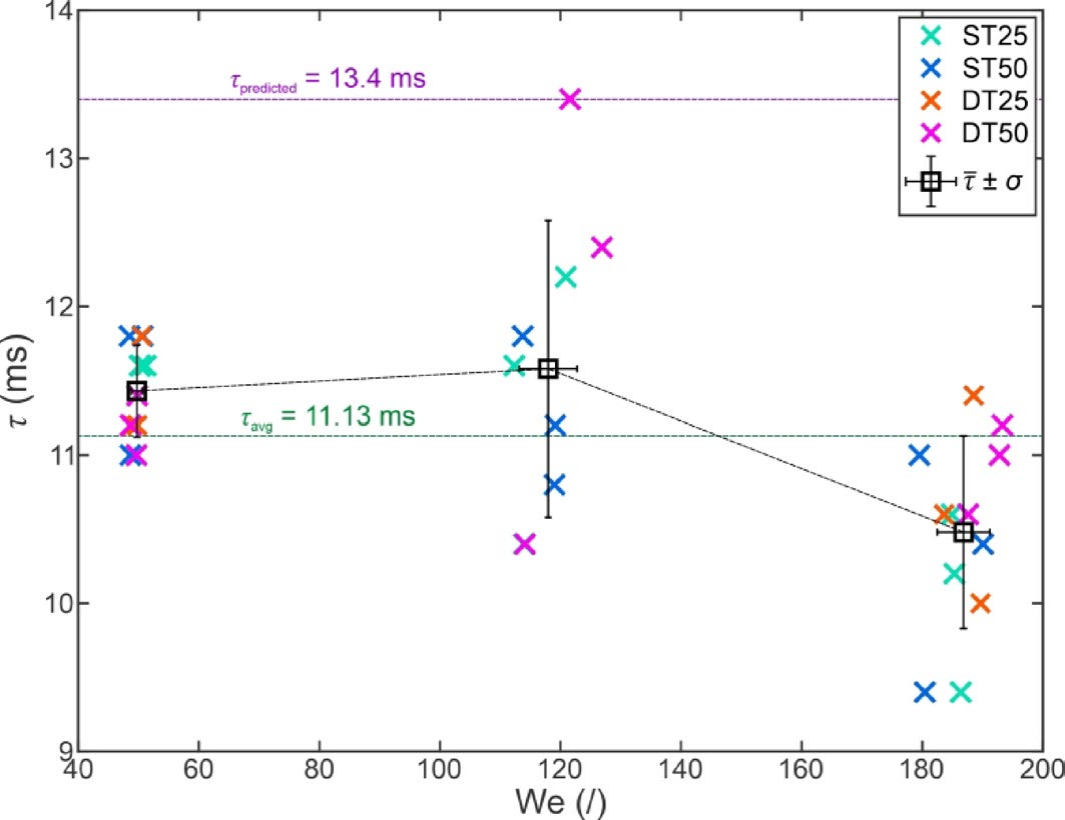
Droplet contact times during the full rebound versus the Weber number on four superhydrophobic surfaces with different morphologies at a surface temperature of 25 °C. For each of the three average Weber numbers, the average contact time is plotted with black squares, including the standard deviation of *τ* and We as error bars. The violet dashed line shows the predicted droplet-surface contact timescale for the given experimental conditions.

Since the duration of the droplet’s contact with the surface significantly influences heat transfer between the droplet and the substrate, an additional analysis of the contact time was performed. Firstly, we compare in Fig. 12 the droplet-surface contact time for the tests which resulted in a full rebound at a surface temperature of 25 °C on all four superhydrophobic surfaces as a function of the Weber number.

It should be noted that no values are provided for the DT25 surface at We ≅ 120 since a full rebound was not observed at this We. The contact times are highly consistent at We ≅ 50 with all values between 11 and 11.8 ms (average value of 11.4 ms, standard deviation of ± 0.3 ms). At We ≅ 120, a much broader scatter of the values is observed and attributed to the gradual transition toward droplet pinning and splashing, resulting in prolonged contact with the surface. Here, a similar average value for contact time was observed (11.6 ms), but with a much higher standard deviation (± 1.0 ms). Finally, the contact time decreases slightly at the higher We ≅ 185, resulting in an average value of 10.5 ± 0.6 ms. The arithmetical average of all measured contact times was 11.13 ms (see green dashed line in Fig. 12). No clear trend regarding the influence of surface properties on the contact time was observed at any of the Weber number values. The observed values broadly agree with established literature values and predictions, which forecast the timescale for the droplet rebound to be approximately 13.4 ms for room-temperature water droplets with a diameter of 2.48 mm (shown in Fig. 12 with a violet dashed line).

Additionally, to confirm our observation, a one-way analysis of variance was performed for four sets of contact time measurements at 25 °C (each set corresponding to one surface) for each of the three Weber numbers. The results are listed in the *Supporting Information* in section S9 (*One-way analysis of variance (ANOVA) for contact times at 25 °C*). Briefly, the surface morphology was found to be an insignificant factor in terms of influencing the droplet-surface contact time.

**Fig. 13:**
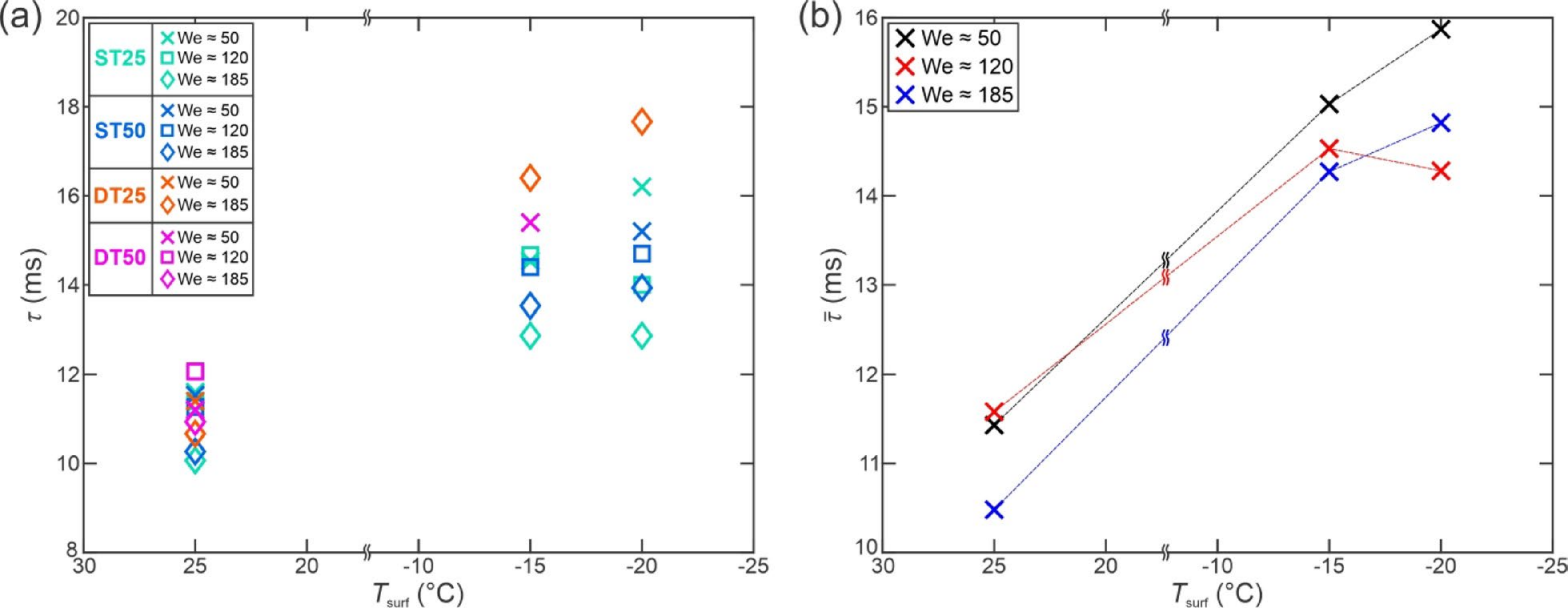
Average droplet contact times for individual surfaces versus the surface temperature for full rebounds (**a**) and overall average contact times for each average We versus the surface temperature (**b**).

Yao et al.^53^ reported an increase in the contact time from 11.9 to 14.2 ms at We = 63 when decreasing the surface temperature from 20 °C to −20 °C, corresponding to an increase of 19.3%. Additionally, Wang et al.^69^ reported an increase in contact time of ~ 31.3% at We = 19 when reducing the surface temperature decreased from − 5 °C to −25 °C. Hence, we also measured the contact times for full rebound on the superhydrophobic surfaces, which were present at −15 °C on most surfaces and at −20 °C on some of them. The results are shown in Fig. 13(a) as the average contact time for each superhydrophobic surface at three different average We and in Fig. 13(b) as the overall average at every surface temperature and We.

A clear trend of increasing droplet contact time is observable with an increase of the average value at We ≅ 50 from 11.4 ms to 15.0 ms and 15.9 ms at −15 °C and − 20 °C, respectively. This represents an increase of 31% and 39%, respectively, agreeing with the reports by Yao et al.^53^ and Wang et al.^69^. A similar trend was observed at We ≅ 120 (+ 26% and + 23% at −15 °C and − 20 °C, respectively) and at We ≅ 185 (+ 36% and + 41% at −15 °C and − 20 °C, respectively). The non-monotonous trend of increasing contact time with decreasing surface temperature at We ≅ 120 can again likely be attributed to the transitional nature of the Weber number, where droplet splashing starts to appear.

As the surface temperature decreases, the temperature of the droplet in contact with the surface during the impact also decreases, resulting in a significant increase in viscosity^70^increasing the frictional and adhesion force magnitude, thus reducing the retraction rate and prolonging the droplet-surface contact time. Specifically, the dynamic viscosity of a droplet increases by a factor of ~ 3.3 times at −15 °C compared to the value at 20 °C, while the surface tension increases by about 7.2%^71^. As shown in the Section"Outcome of the droplet impact as a function of surface temperature and the Weber number"and Fig. 8, the droplets stop to exhibit a (full) rebound if the surface temperature is decreased further (i.e., to −25 °C or less in our case), meaning that the onset of freezing appears due to the sufficiently long droplet-surface contact. It is, however, possible that such low surface temperatures also result in the frost formation on the test surface despite its superhydrophobicity and the generally low relative humidity in the test chamber, leading to early freezing of the impacting droplet. This is analyzed in detail in the next section.

Yu et al.^71^ have stipulated that condensation in the surface microstructure may increase the contact time, showing that supersaturation (the ratio between the vapor pressure and the saturation pressure) of 3.8 already appears with the surface at 0 °C and the droplet at 20 °C. Importantly, the supersaturation increases significantly with decreased surface temperature, reaching 12.2 at −15 °C and 28.9 at −25 °C. Therefore, it is likely that condensation and freezing of the vapor stemming from the droplet’s immediate surroundings contribute to increased contact times and a higher likelihood of a partial rebound or complete adhesion with decreasing surface temperatures. Yu et al. have observed droplet rebounds with contact times up to a maximum value of ~ 25 ms, above which adhesion of the droplet was universally noted. This agrees with the observation of the present study, where the maximal contact times for a successful full rebound reached 19.4 ms, while partial rebounds (not shown in Figs. 12 and 13) generally exhibited contact times of > 20 ms.

### Effect of humidity and frosting on droplet impact outcome

**Fig. 14:**
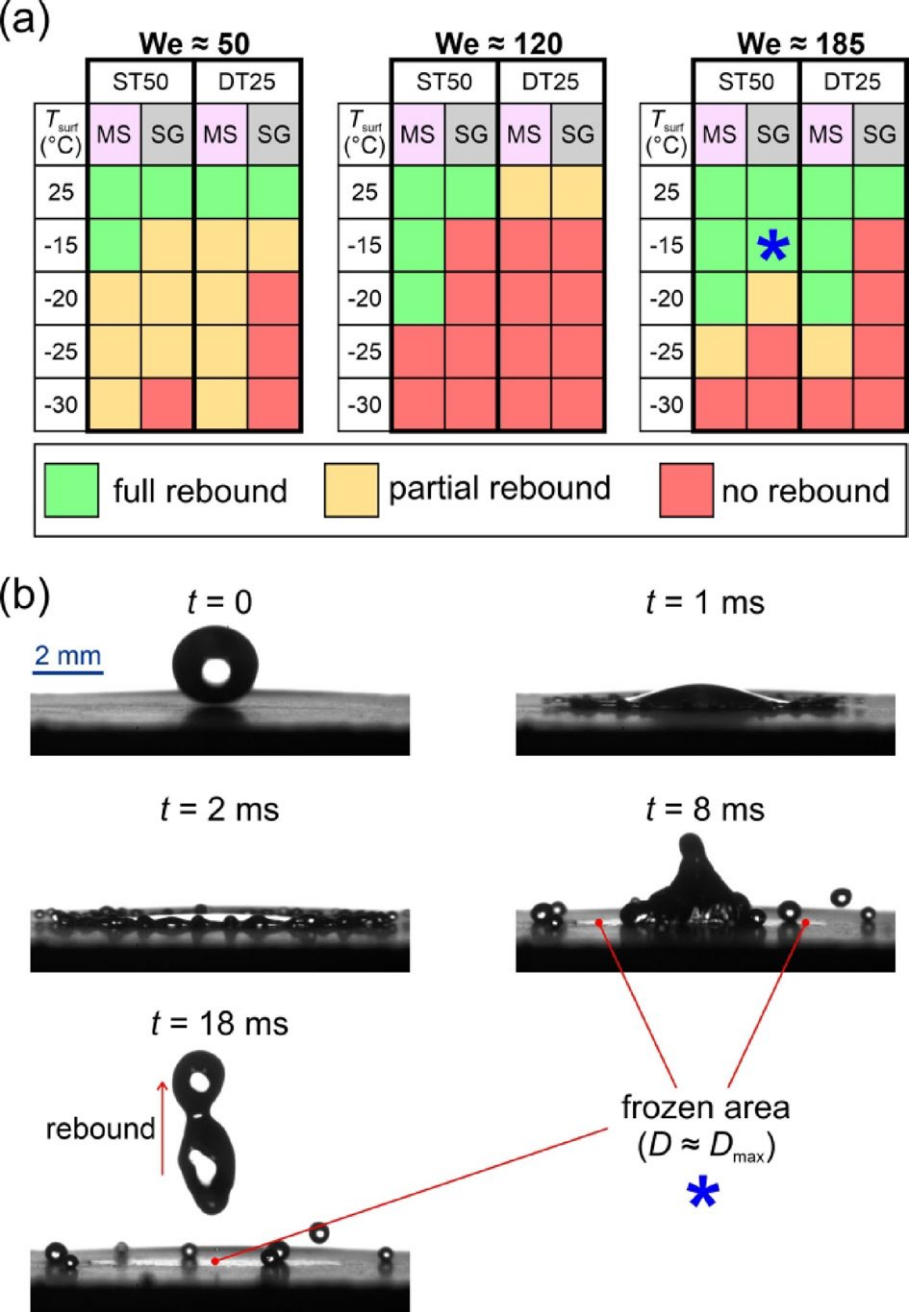
The impact of desiccant on the droplet impact outcome for different surface temperatures at three Weber numbers (**a**) and snapshots of the droplet rebound on the ST50 surface at We ≅ 185 and − 15 °C with silica gel as the desiccant (**b**). MS denotes the use of molecular sieves (RH < 7%) while SG denotes the use of silica gel (RH 15–20%).

Higher humidity levels in the experimental chamber can result in frost growth and accumulation on the test surface, which significantly affects the spreading, rebounding, and freezing behavior of an impacting droplet but at the same time better resemble typical environmental conditions. For example, air at 23 °C (average temperature during our experiments) with 20% relative humidity (achieved, for example, using silica gel) has a dew point at approx. −1 °C, while air at the same temperature but with 7% relative humidity (the maximum achieved with molecular sieves, during most of the experiments) has a dew point of approx. −15 °C. To study the effect of humidity and frost formation on the impact freezing dynamics, we performed additional experiments using the ST50 and the DT25 surface. Outcomes of the droplet impact in experiments with higher and lower humidity are shown in Fig. 14(a) in the same manner they were previously presented in Fig. 8. Additionally, snapshots of the impacts are available in the *Supporting Information* in section S10 (*Snapshots of droplet impacts at two different relative humidity levels*). Specifically, the droplet impact outcome is compared for a relative humidity under 7% (desiccant: molecular sieves, abbreviation “MS”) and a relative humidity between 15 and 20% (desiccant: silica gel, abbreviation “SG”).

The results reveal that both for the surface with shallow microchannels (i.e., ST50) and the surface with deep irregular microchannels (i.e., DT25), higher humidity in the test chamber shifts the failure of the (full) rebound to higher surface temperatures (i.e., lower subcooling below the freezing point). At We ≅ 50, the droplet failed to fully rebound at higher humidity at all subzero temperatures. Also, it exhibited full adhesion with no rebound at −20 °C or below on the DT25 surface, where full adhesion was not previously noted at any temperature during the experiments with low humidity. At We ≅ 120, no rebound with full adhesion was observed on the ST50 surface at all subzero temperatures, while at low humidity, the same surface provided a full rebound down to −20 °C. The DT25 surface failed to provide any full rebounds at We ≅ 120 at both humidity levels. Finally, at We ≅ 185, the DT25 surface provided a full rebound down to −20 °C and a partial rebound at −25 °C at a low humidity but failed to rebound any droplets at a higher humidity. At this Weber number, an interesting phenomenon was observed on the ST50 surface. While its rebound performance was again worsened at a higher humidity compared to the low humidity tests, a full droplet rebound with deposition of an ice layer was observed at −15 °C (denoted with a blue asterisk). This phenomenon was repeatedly observed in three tests on different parts of the ST50 sample’s surface with an analysis of the high-speed snapshots shown in Fig. 14(b). When the droplet flattened during the spreading phase, freezing occurred under the lamella, and an ice disc with a diameter roughly matching the droplet’s maximum spreading diameter was deposited onto the surface. However, the sufficient inertial energy during the retraction, coupled with a low surface subcooling below the freezing temperature, enabled a full rebound of the remaining part of the droplet. This phenomenon was not observed on the DT25 surface or during any of the other tests.

Overall, higher humidity during the experiments universally resulted in a degraded ability of the surfaces to rebound water droplets. This implies that humidity should be stringently controlled during experiments to ensure compatibility between different samples/surfaces and with other studies, which are predominantly performed at low humidity levels (generally below 15%). On the other hand, low humidity tests might not fairly evaluate the surface’s ability to operate under realistic conditions, where higher humidity levels could result in significantly worse performance than predicted during the laboratory tests at very low humidity values.

**Fig. 15:**
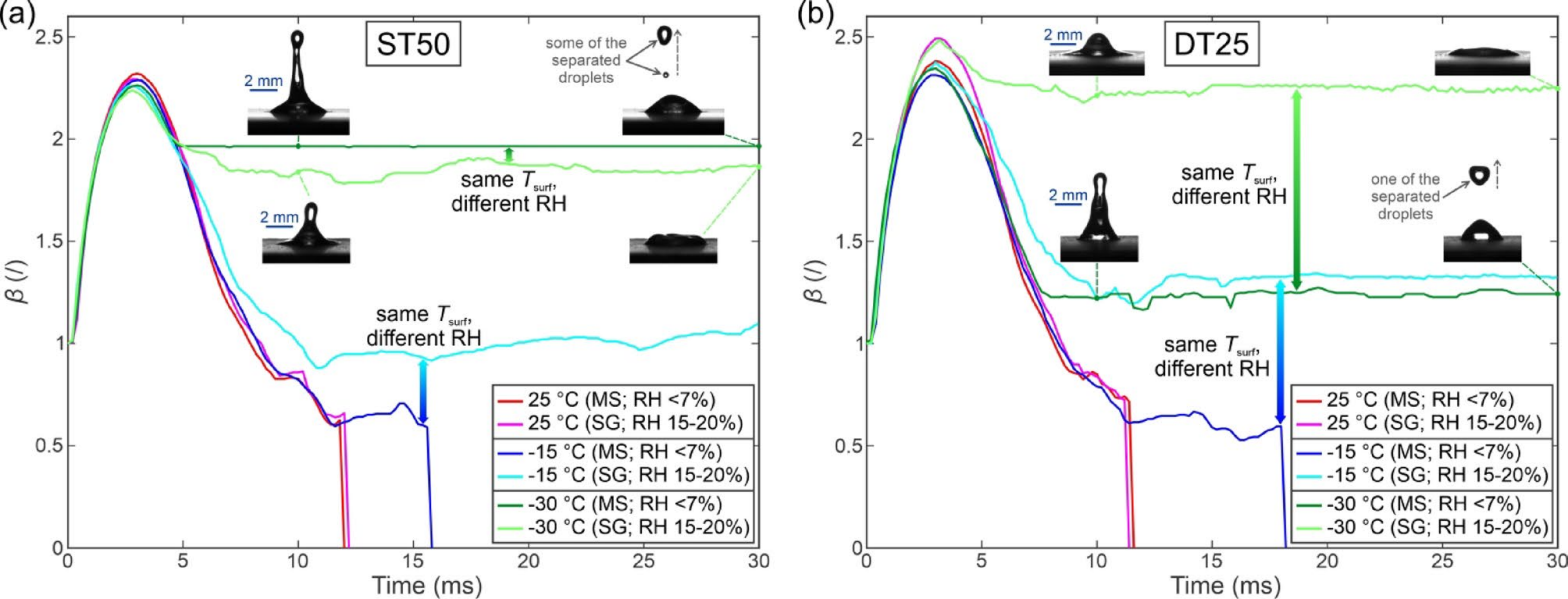
The effect of relative humidity of the air on the temporal evolution of the spreading factor on (**a**) ST50 surface and (**b**) DT25 surface for three surface temperatures. MS denotes the use of molecular sieves (RH < 7%) while SG denotes the use of silica gel (RH 15–20%).

To compare the effect of humidity and consequent frost accumulation on the spreading of the droplets, an analysis of the spreading factor’s temporal evolution is shown in Fig. 15(a) for the ST50 surface at three temperatures and two relative humidity levels and in Fig. 15(b) for the DT25 surface for the same set of temperatures and humidity levels. In both cases, data for We ≅ 50 is shown. As expected, no significant differences between the tests at different humidity are noticeable for both surfaces at the room temperature case with the surface at 25 °C, where no condensation or frost accumulation on the surface can occur. At −15 °C, the most significant differences appear as the droplets exhibit partial adhesion at a higher humidity while a full rebound is still present at a lower humidity (e.g., on surface ST50). At an even lower surface temperature (−30 °C), full adhesion with no rebound happens at higher humidity. In contrast, at least a partial rebound occurs at a lower humidity value, as the inset high-speed snapshots show. Again, the maximum diameter of the droplet was tracked from the moment of first contact with the surface to the moment of rebound (if it was present), hence, a sharp drop of *β* to zero is evident for full rebounds.

Gao et al.^72^ previously showed that surface frosting due to elevated environmental humidity significantly affects both the motion of the three-phase contact line and the icephobic performance on various non-hydrophobic surfaces. The spreading and retraction of the droplet are constrained by the combined effects of surface supercooling and frosting, resulting in a decline in the surface’s icephobic properties. Moreover, at a surface temperature of −30 °C, individual satellite droplets were observed on the frosted surface, attributed to the roughness created by the frost forming pockets that trap the initial droplets. In our case, the opposite behavior was observed with the ejection of separated smaller droplets or a partial rebound of the entire droplet observed under lower humidity with little-to-no frosting. On the other hand, no such phenomenon was observed at a higher humidity where some frosting was present, and the droplet was pinned in place. We can conclude that the interaction of a droplet with a cold, frosted surface results in higher dissipation of the droplet’s kinetic energy and, together with possible freezing of the liquid at the solid/liquid interface during the receding phase, reduces its level enough for only horizontal oscillations to appear but no vertical stretching and necking (that would otherwise take place in the departure phase during a rebound). This is also confirmed in Figs. S15-S17 in the *Supporting Information* in section S10, where snapshots of the droplet impact are compared on the ST50 and the DT25 surface for two different humidity levels for surface temperatures of −15 °C and − 30 °C.

Guo et al.^28^ investigated the bouncing regimes of supercooled water droplets impacting superhydrophobic surfaces at different surface temperatures and environmental humidity values. On a flat superhydrophobic surface, the authors observed a successful full rebound of the droplet down to a surface temperature of −30 °C at a relative humidity of 7% for Weber numbers between 20.4 and 122.1 (with only one exception at the highest We and lowest surface temperature). When repeating the experiments at a higher humidity of 15%, a transition from a full rebound to full adhesion was observed when reducing the surface temperature from − 20 °C to −25 °C, regardless of the Weber number. Similar observations were made on a post-array superhydrophobic surface, where the transitions between bouncing regimes occurred one 5 K temperature step earlier (e.g., at −15 °C instead of at −20 °C). Furthermore, the pancake bouncing mechanism failed due to the droplet adhesion caused by a frost layer on a sufficiently subcooled surface. A scale analysis indicated that the frost between the posts reduces the capillary energy stored during the downward penetration, resulting in the failure of the pancake bouncing. Despite the general absence of the pancake rebound mechanism on our surfaces, the observations by Guo et al. agree well with our observations and previously provided explanations regarding the effect of frost on the surface on the transition between droplet rebound outcomes.

## Conclusions

This study investigated the spreading, rebound, and freezing behavior of water droplets on superhydrophobic laser-textured aluminum surfaces, focusing on the effects of surface morphology. Superhydrophobic test surfaces (*θ*_*A*_ > 163° and contact angle hysteresis < 2°) with deep (~ 30 μm) or shallow (~ 3 μm) structures were fabricated and included either evenly spaced topographical elements (i.e., microchannels) or irregular features. Droplet impact tests were performed at Weber numbers between 50 and 185 while the surface temperature was varied between 25 °C and − 30 °C. Finally, the effect of environmental humidity was evaluated by comparing the results obtained at a relative humidity < 7% or 15–20%.

The following main conclusions were reached.

1. The maximum spreading factor (*β*_max_) at room temperature was unaffected by the surface morphology. At the lowest surface temperature (−30 °C), *β*_max_ decreased slightly compared to room temperature with a maximum reduction of 9.5% at We ≅ 185.
2. Comparison of the measured maximum spreading factor at room temperature with predictions of eight literature-sourced models revealed the best fit is provided by the model proposed by Asai et al.^12^ (mean average percentage error (MAPE) of 9.5%), followed by models by Mao et al.^15^ (MAPE: 11.4%) and Roisman^13^ (MAPE: 11.8%).
3. Analysis of droplet impact outcomes (full rebound, partial rebound, or no rebound with full adhesion of the droplet) revealed that the two surfaces with shallow features on average provided the most favorable water-repellent properties at subcooled surface temperatures, which we attribute to the lower value of the solid-liquid contact fraction.
4. Surprisingly, the poorest rebound performance for all surfaces was obtained at the We ≅ 120 where partial rebounds or full adhesion were more common than at We ≅ 50 or We ≅ 185. High-speed imaging confirmed the transition between the regular rebound and the splashing regime at We ≅ 120, which was identified as the main factor for decreased full droplet rebound performance.
5. An average contact time of 11.1 ms was obtained across all four tested superhydrophobic surfaces at room temperature, again with no clear trend regarding the influence of surface properties at any We. The average contact times increased between 23% and 41% when the surface temperature was decreased to −20 °C. Partial droplet adhesion was observed when the contact times exceeded 20 ms.
6. Tests at a higher relative humidity revealed that the unfavorable outcomes of droplet impact (i.e., partial rebound or no rebound at all) appear at higher temperatures compared to the tests at lower humidity, which is caused by frost on the surface, which causes higher dissipation of the droplet’s kinetic energy. Together with possible freezing of the liquid at the solid/liquid interface during the receding phase, this reduces the droplet’s energy enough for only horizontal oscillations to appear but no vertical stretching and necking that would otherwise take place in the departure phase during a rebound.

Within the range of tested variations, the surface microstructure was shown to only affect the outcome of the droplet’s impact with a subcooled surface in terms of the (in)existence of a rebound, where the shallow microstructures were found to be preferable to deep surface features, which is ascribed to the lower actual surface area of the former and thus a lower roughness factor compared to the surfaces with deep features. Considering commonly observed lower ice adhesion values on surfaces with lower roughness, this opens a new direction for the future research and development of water-repellant and icephobic superhydrophobic surfaces with minimal roughness and micrometric or submicrometric features.

## Supplementary Information

Below is the link to the electronic supplementary material.

Supplementary Material 1

## Data Availability

The authors confirm that the data supporting the findings of this study are available either within the article, the Supporting Information file, or from the corresponding author upon request.

## Abbreviations

CAH: Contact angle hysteresis (°)
*c*_*s*_: Speed of sound in liquid (m s^-1^)
*D*_0_: Droplet diameter before impact (m)
*D*_b_: Focused beam diameter (m)
*D*: Droplet width at a given moment (m)
*F*: Average fluence (J cm^-2^)
*f*: Pulse frequency (Hz)
*h*: Microchannel height (μm)
*k*: Empirical constant
M^2^: Laser beam quality
Oh: Ohnesorge number
*P*: Average laser power (W)
*p*_*C*_: Capillary pressure (Pa)
*p*_*D*_: Dynamic pressure (Pa)
*p*_*WH*_: Water hammer pressure (Pa)
*r*: Roughness factor
Re: Reynolds number
*Sa*: Arithmetical mean height of a surface (μm)
*T*: Temperature (K; °C)
*t*: Time (s)
*u*_0_: Droplet velocity before impact (m s^-1^)
*w*: Microchannel width (μm)
We: Weber number

## Greek letters

*β*: Spreading coefficient
*θ*: Static contact angle (°)
*θ*_A_: Advancing contact angle (°)
*θ*_R_: Receding contact angle (°)
*θ*_Y_: Young contact angle (°)
*λ*: Laser wavelength (ns)
*μ*: Dynamic viscosity (Pa s)
*ρ*: Density (kg m^-3^)
*σ*: Surface tension (N m^-1^)
*τ*: Contact time (s)
*Φ*: Solid-liquid contact area
*φ*: Solid fraction

## Subscripts

final: Final
L: Liquid
max: Maximum
surf: Surface
