# Supplementary material for: Surface morphology effects on droplet spreading and rebound dynamics on subcooled superhydrophobic surfaces
Source: Sci Rep. 2025 Aug 12;15:29530. doi: 10.1038/s41598-025-14634-4 (PMC12343892; doi:10.1038/s41598-025-14634-4)
Supplement: Supplementary file 1 — Supplementary Material 1 [file 41598_2025_14634_MOESM1_ESM.pdf]

Supporting Information

Surface morphology effects on droplet spreading and rebound dynamics on subcooled superhydrophobic surfaces

Matic Može [a,*], Yuheng Shang [b], Samo Jereb [a], Nina Kovač [c,d], Miha Štucin [a], Tim Štrus [a], Peter Rodič [c], Nina Kovač [c], Matevž Zupančič [a], Maria Rosaria Vetrano [b], Iztok Golobič [a]

[a] *University of Ljubljana, Faculty of Mechanical Engineering, Aškerčeva c. 6, SI-1000 Ljubljana, Slovenia*

[b] *KU Leuven, Department of Mechanical Engineering, Division of Applied Mechanics and Energy Conversion (TME), B-3001 Leuven, Belgium*

[c] *Jožef Stefan Institute, Department of Physical and Organic Chemistry, Jamova c. 39, SI-1000 Ljubljana, Slovenia*

[d] *Jožef Stefan International Postgraduate School, Jamova c. 39, SI-1000 Ljubljana, Slovenia*

*Corresponding author: matic.moze@fs.uni-lj.si; +386 1 4771 309

Table of Contents

S1. SEM images of the reference surface 3
S2. Roughness metrics of the test surfaces 4
S3. Maximum spreading factor versus the observation direction relative to the laser-made channels 6
S4. Maximum spreading factor versus the surface temperature 8
S5. Statistical evaluation of different maximum spreading factor models 10
S6. Snapshots of droplet impacts at various temperatures and We ≅ 50 11
S7. Snapshots of droplet impacts at various temperatures and We ≅ 120 14
S8. Snapshots of droplet impacts at various temperatures and We ≅ 185 17
S9. One-way analysis of variance (ANOVA) for contact times at 25 °C 20
S10. Snapshots of droplet impacts at two different relative humidity levels 21
References 25

S1. SEM images of the reference surface

Figure S1 shows a compilation of SEM images taken on the untreated reference surface (“R”). It is noticeable that any distinct surface morphology is absent, matching the measured low surface roughness. No SEM analysis was performed on the hydrophobized reference surface (“R-H”) as its morphology matched that of the untreated surface “R” and the applied coating is a monolayer only a few nanometers thick, making it unobservable with SEM imaging.

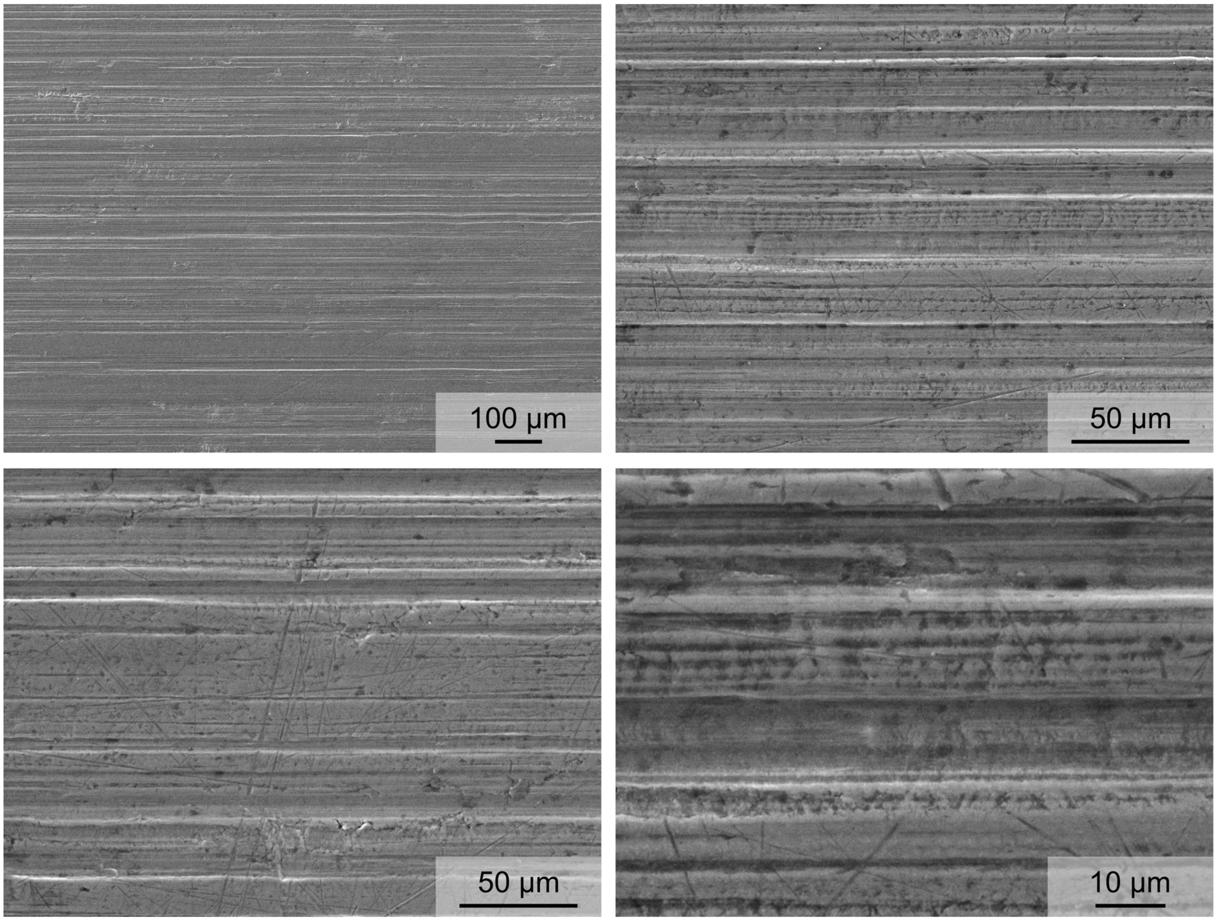

Figure S1. SEM images of the untreated reference surface.

S2. Roughness metrics of the test surfaces

Roughness parameters recorded on the laser-textured test surfaces using a digital microscope (Keyence VHX-6000) are given in Table S1. Directional parameters (Ra and Rz) were measured perpendicular to the direction of laser texturing (*i.e.*, across the grooves). Sz denotes the maximum height of the profile and is defined as the sum of the largest peak height value and the largest pit depth value within the defined area. It represents the areal extension of the Rz metric commonly used to evaluate line profiles.

Table S1. Roughness parameters measured on the laser-textured surfaces.

Surface	Ra (µm)	Rz (µm)	Sz (µm)
ST25	1	6	13
ST50	2	7	14
DT25	5	18	32
DT50	10	35	45

Additionally, the surface topography of textured surfaces was evaluated at three randomly selected spots using a stylus contact profilometer (Bruker DektakXT with a 2 μm tip and operated in soft-touch mode with a force of 1 mN). Measurements were conducted over an area of 0.75 × 0.75 mm, with a vertical analysis range of 65.5 μm and a vertical resolution of 0.167 μm/point. The data collected were analyzed using TalyMap Gold 6.2 software. The results are shown as 3D images with the same vertical axis scalebar in Fig. S2.

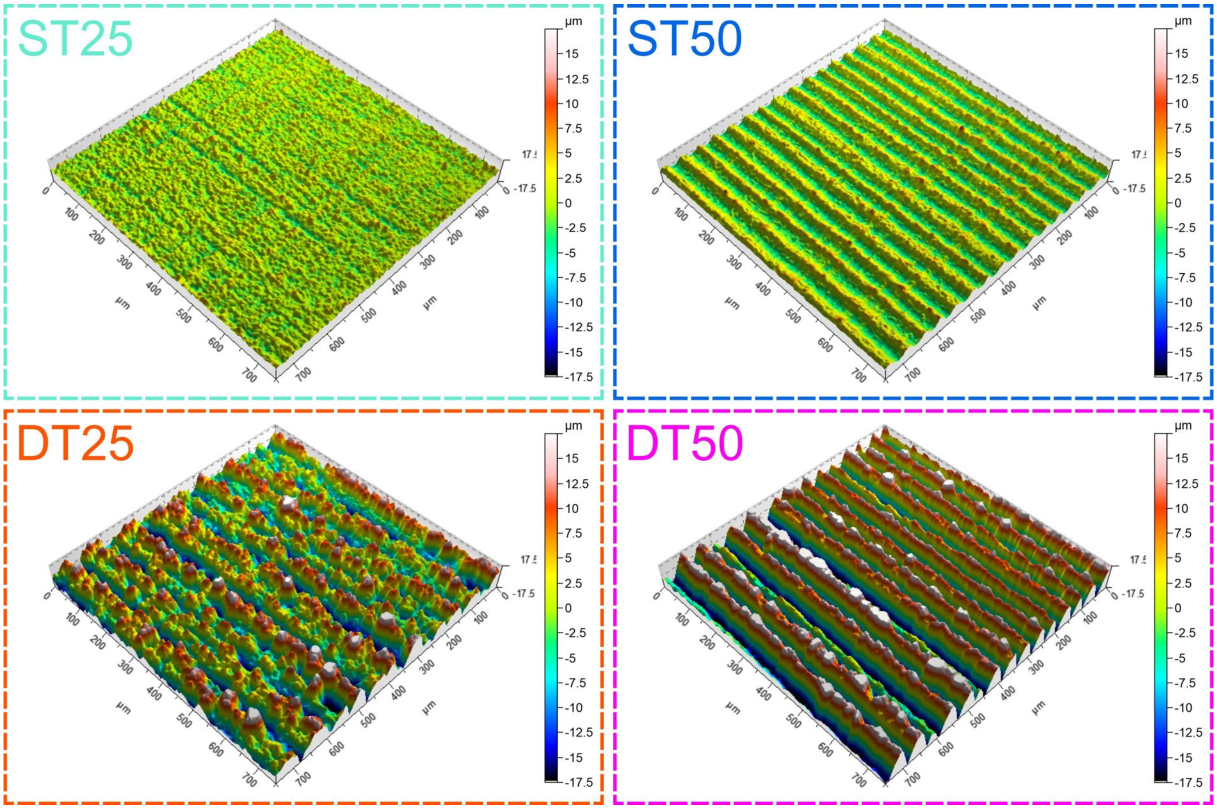

Figure S2. 3D topographical images of the four laser-textured test surfaces.

S3. Maximum spreading factor versus the observation direction relative to the laser-made channels

An analysis to compare the maximum spreading factor of the droplet during impact by observing the spreading either parallel to the laser-made channels and perpendicular to them was performed at We = 89.9 ± 0.5. The results are provided in Table S2 as arithmetic means of five measurements alongside the corresponding standard deviation.

Table S2. Maximum spreading factor at We = 89.9 ± 0.5 for different observation directions relative to the direction of the laser-made channels.

**Surface**	**β_{max} (/)**	
	**Parallel to the channels**	**Perpendicular to the channels**
ST25	2.956 ± 0.077	2.930 ± 0.135
ST50	2.823 ± 0.052	2.757 ± 0.023
DT25	3.171 ± 0.134	2.916 ± 0.094
DT50	3.053 ± 0.047	2.812 ± 0.008

Furthermore, a one-way repeated measures ANOVA was performed to compare the variation of the results within each group of measurements (*i.e.*, five measurements for one direction of channels on a selected surface) and between the two groups of measurements (*i.e.*, between the two observation directions relative to the channels). The results are provided in Table S3.

The results indicate that the *p*-value is smaller than the typical threshold value for significance ($p < 0.05$) only on the two surfaces with deep textures (DT). On the shallow texture (ST) surfaces, the value is above the threshold, indicating that the differences in droplet spreading either parallel or perpendicular to the channels are statistically insignificant. Hence, all high-speed imaging in the study was performed in the same orientation, with the droplet spreading observed parallel to the laser-made channels.

Table S3. One-way repeated measures ANOVA results for droplet spreading tests at We = 89.9 ± 0.5 for different observation directions relative to the direction of the laser-made channels.

**Surface**	**Mean Square (MS)**			***F* statistic**	***p*-value**
	**Between groups**	**Inside groups**	**Error**		
ST25	0.0017	0.0121	0.008	0.216	0.666
ST50	0.011	0.0016	0.0015	7.28	0.054
DT25	0.1621	0.0134	0.0068	23.8	0.006
DT50	0.1452	0.0011	0.0009	163	< 0.001

S4. Maximum spreading factor versus the surface temperature

Figures S3-S5 show the maximum spreading factor versus the surface temperature for the three average Weber numbers used in the study.

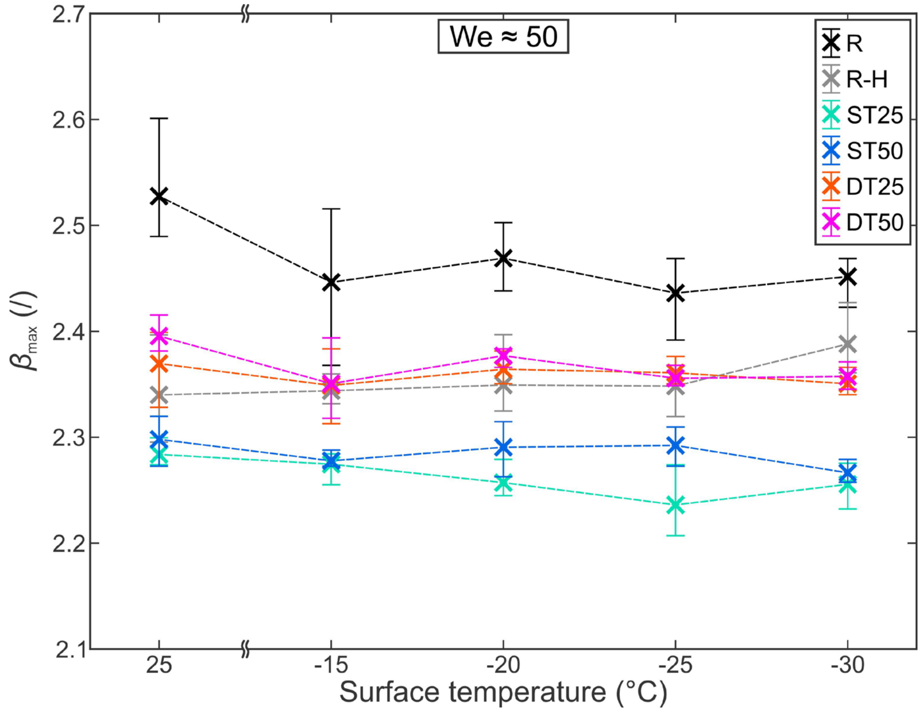

Figure S3. Maximum spreading factor versus the surface temperature at We ≅ 50.

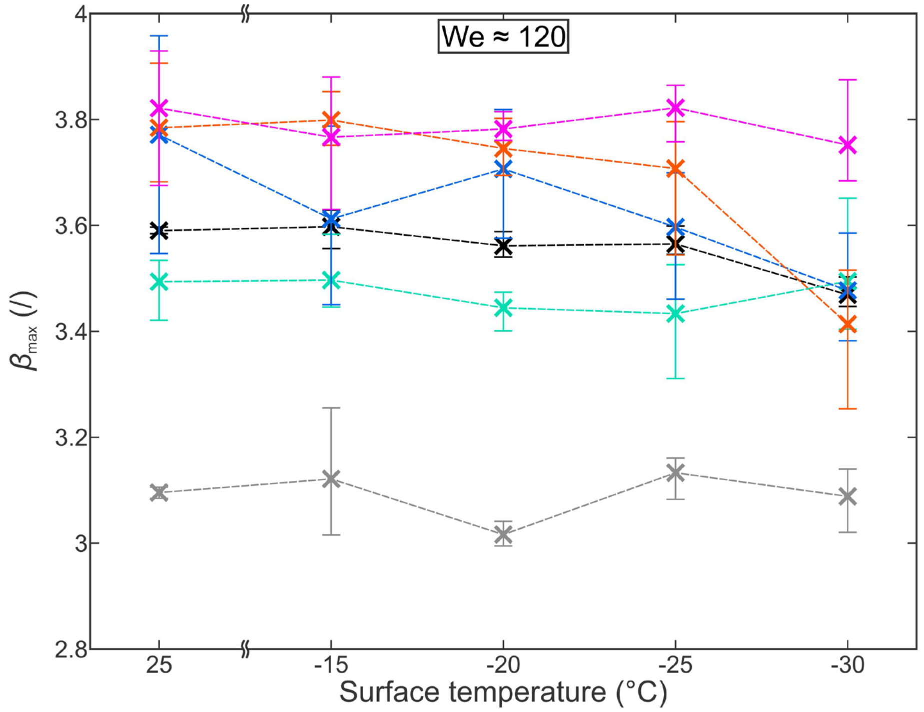

Figure S4. Maximum spreading factor versus the surface temperature at We ≅ 120.

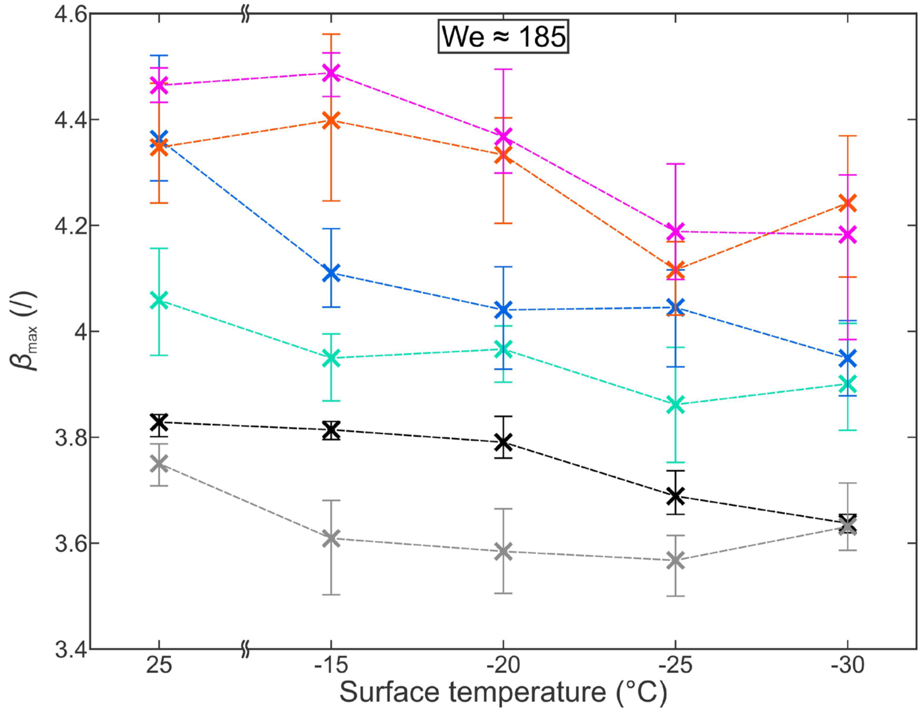

Figure S5. Maximum spreading factor versus the surface temperature at We ≅ 185.

S5. Statistical evaluation of different maximum spreading factor models

Table S4 shows an evaluation of the goodness of fit of the eight models compared in this study. Common statistical metrics are used to evaluate how well model predictions match the original (measured) values. Briefly, the *mean absolute error* (MAE) measures the average magnitude of the errors between predicted and measured values, without considering their direction. The *mean squared error* (MSE) measures the average of the squares of the errors, giving more weight to larger errors. The *root mean squared error* (RMSE) is the square root of MSE, providing an error metric in the same units as the original data. The *mean absolute percentage error* (MAPE) expresses errors as a percentage of the actual values, providing a normalized measure of prediction accuracy. Finally, the *R-squared* metric (R^2; *coefficient of determination*) measures the proportion of the variance in the dependent variable that is predictable from the independent variables. For MAE, MSE, RMSE, and MAPE, values closer to zero indicate a better fit, while the R^2 should be as close to one as possible.

Table S4. Statistical metrics of the maximum spreading factor fit achieved with various models.

**Author(s)**	**MAE**	**MSE**	**RMSE**	**MAPE**	**R^2**
Jones [1]	0.62	0.45	0.67	20.0%	0.255
Asai et al. [2]	0.28	0.10	0.31	9.5%	0.839
Roisman [3]	0.34	0.16	0.39	11.8%	0.744
Scheller & Bousfield [4]	0.44	0.30	0.55	16.2%	0.510
Chandra & Avedisian [5]	0.82	0.93	0.97	27.1%	-0.529
Mao et al. [6]	0.42	0.29	0.54	11.4%	0.530
Aksoy et al. [7]	0.40	0.22	0.47	14.0%	0.641
Pasandideh-Fard et al. [8]	0.48	0.29	0.54	13.2%	0.588

S6. Snapshots of droplet impacts at various temperatures and We ≅ 50

Figures S6-S8 show a comparison of droplet impacts at Weber number of 50 ± 2 on all six surfaces at three selected temperatures, namely 25 °C, -15 °C, and -30 °C.

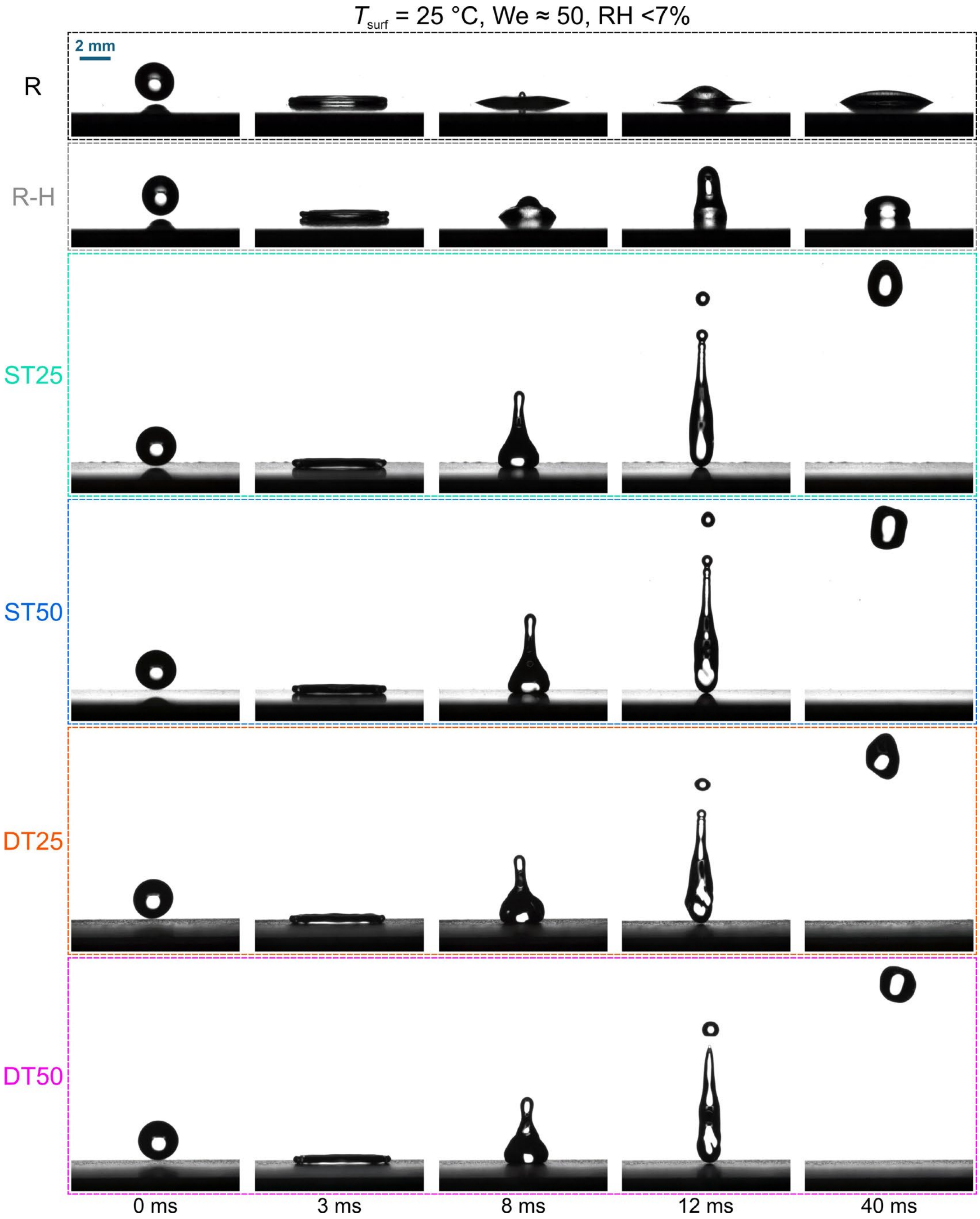

Figure S6. Droplet impacts at a surface temperature of 25 °C and We ≅ 50.

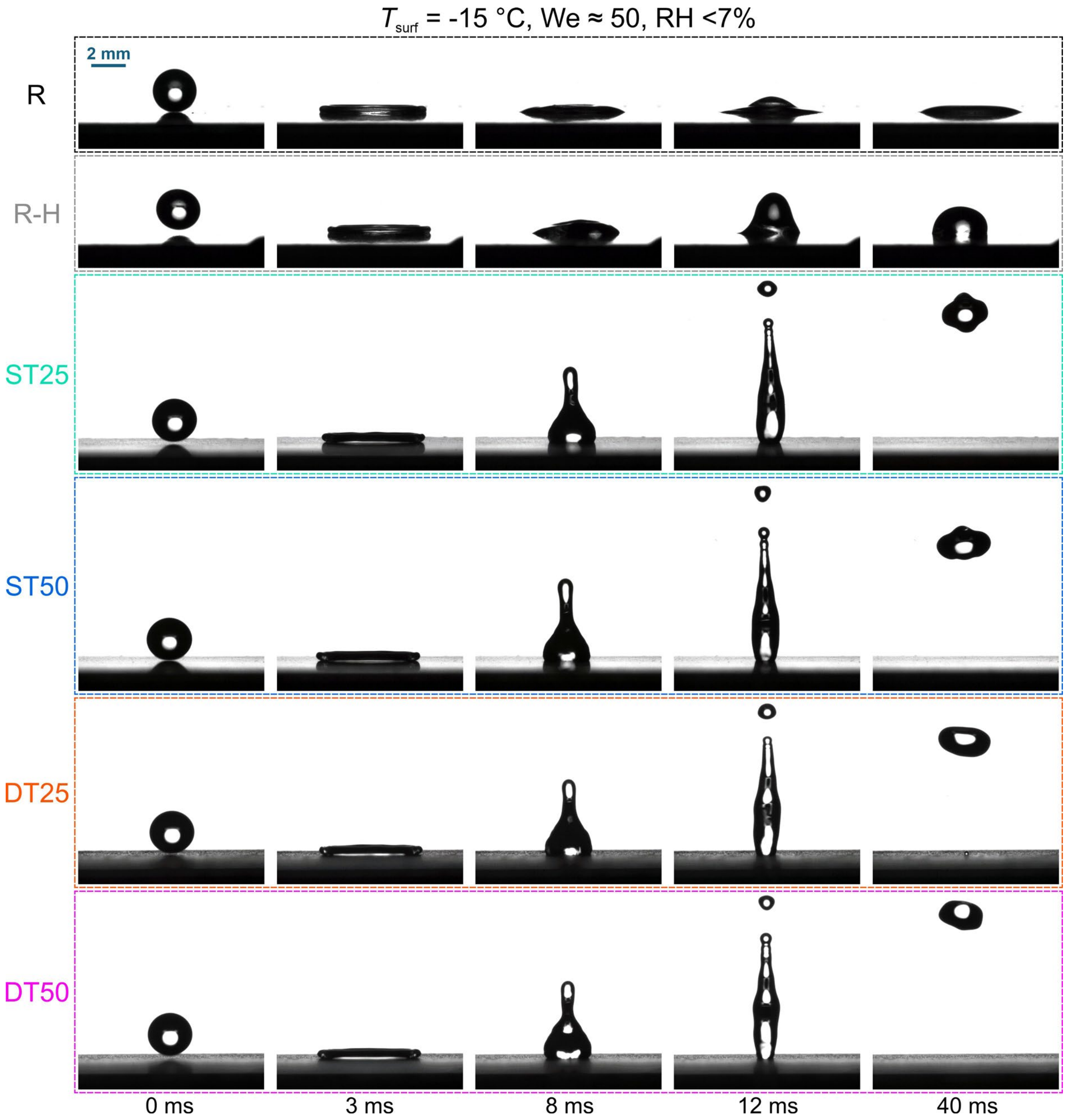

Figure S7. Droplet impacts at a surface temperature of -15 °C and We ≅ 50.

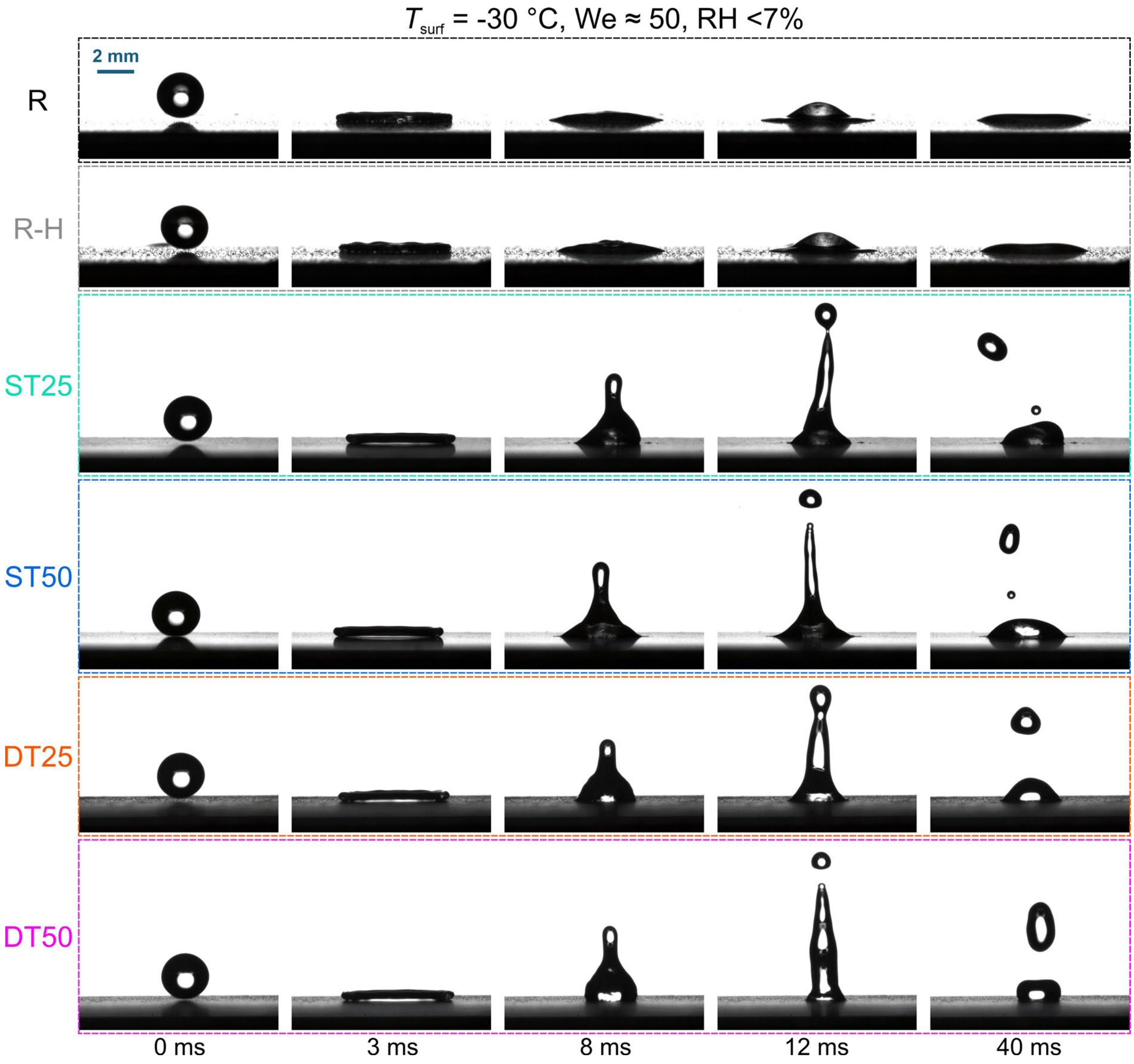

Figure S8. Droplet impacts at a surface temperature of -30 °C and We ≅ 50.

S7. Snapshots of droplet impacts at various temperatures and We ≅ 120

Figures S9-S11 show a comparison of droplet impacts at Weber number of 120 ± 6 on all six surfaces at three selected temperatures, namely 25 °C, -15 °C, and -30 °C.

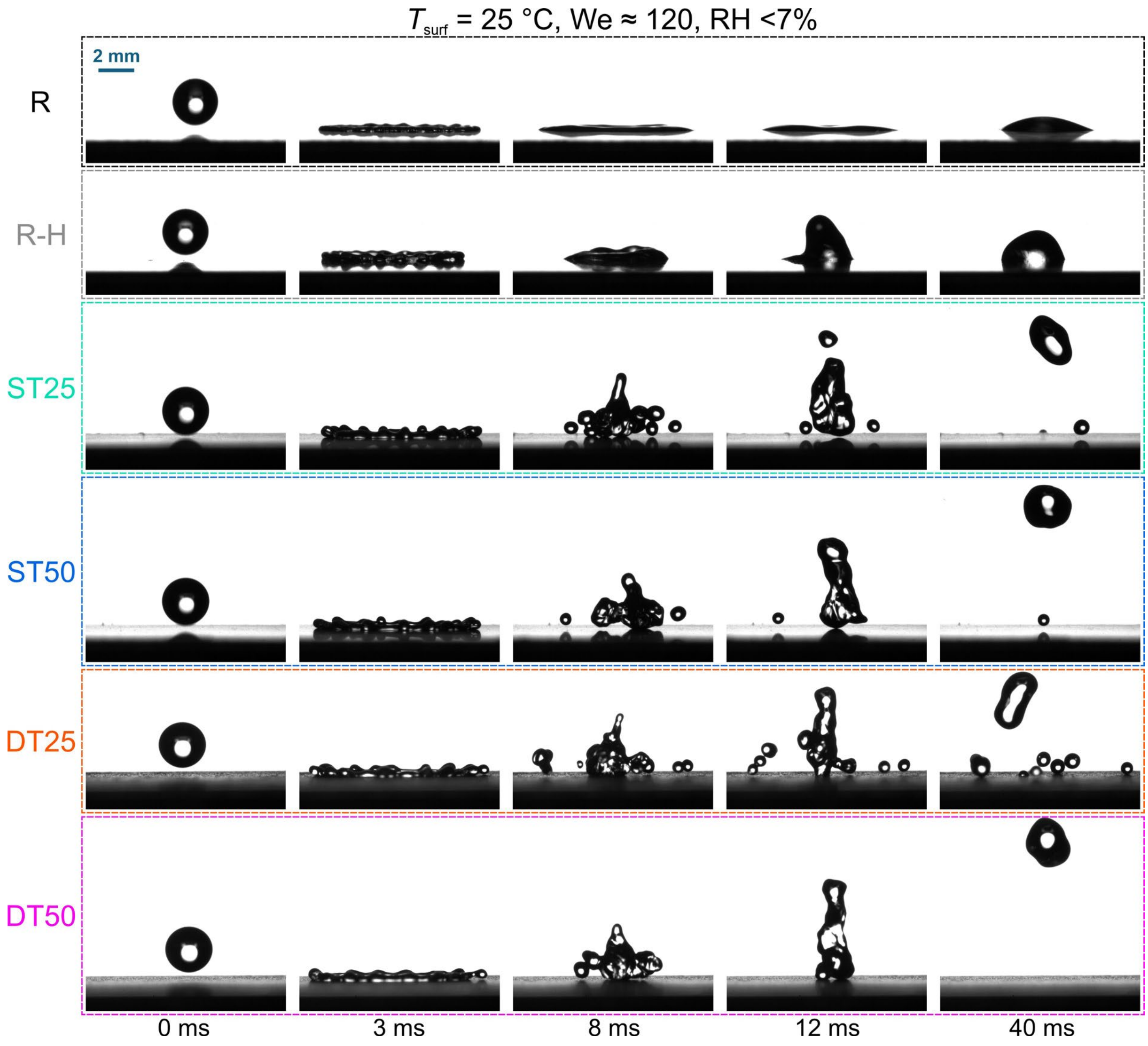

Figure S9. Droplet impacts at a surface temperature of 25 °C and We ≅ 120.

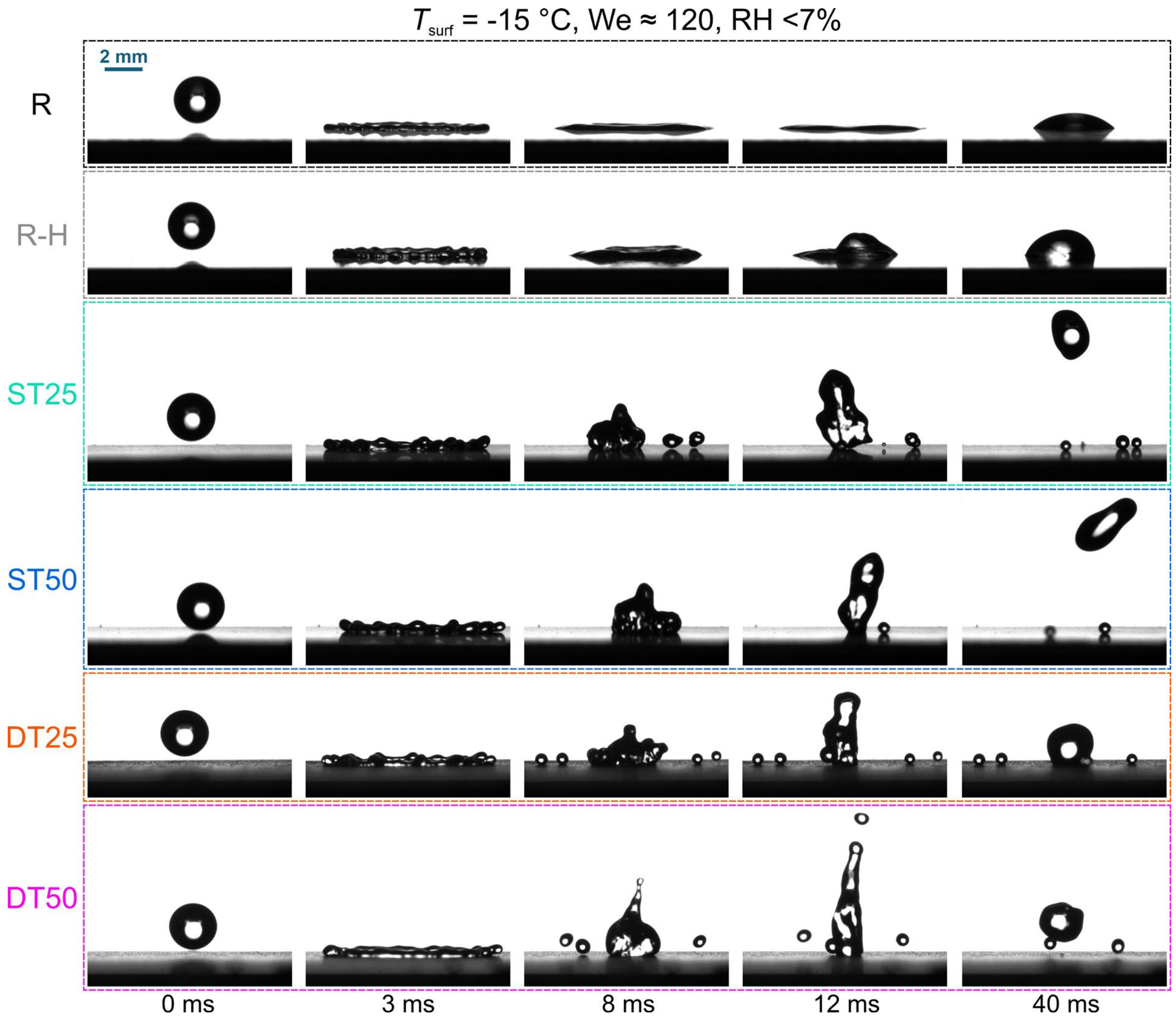

Figure S10. Droplet impacts at a surface temperature of -15 °C and We ≅ 120.

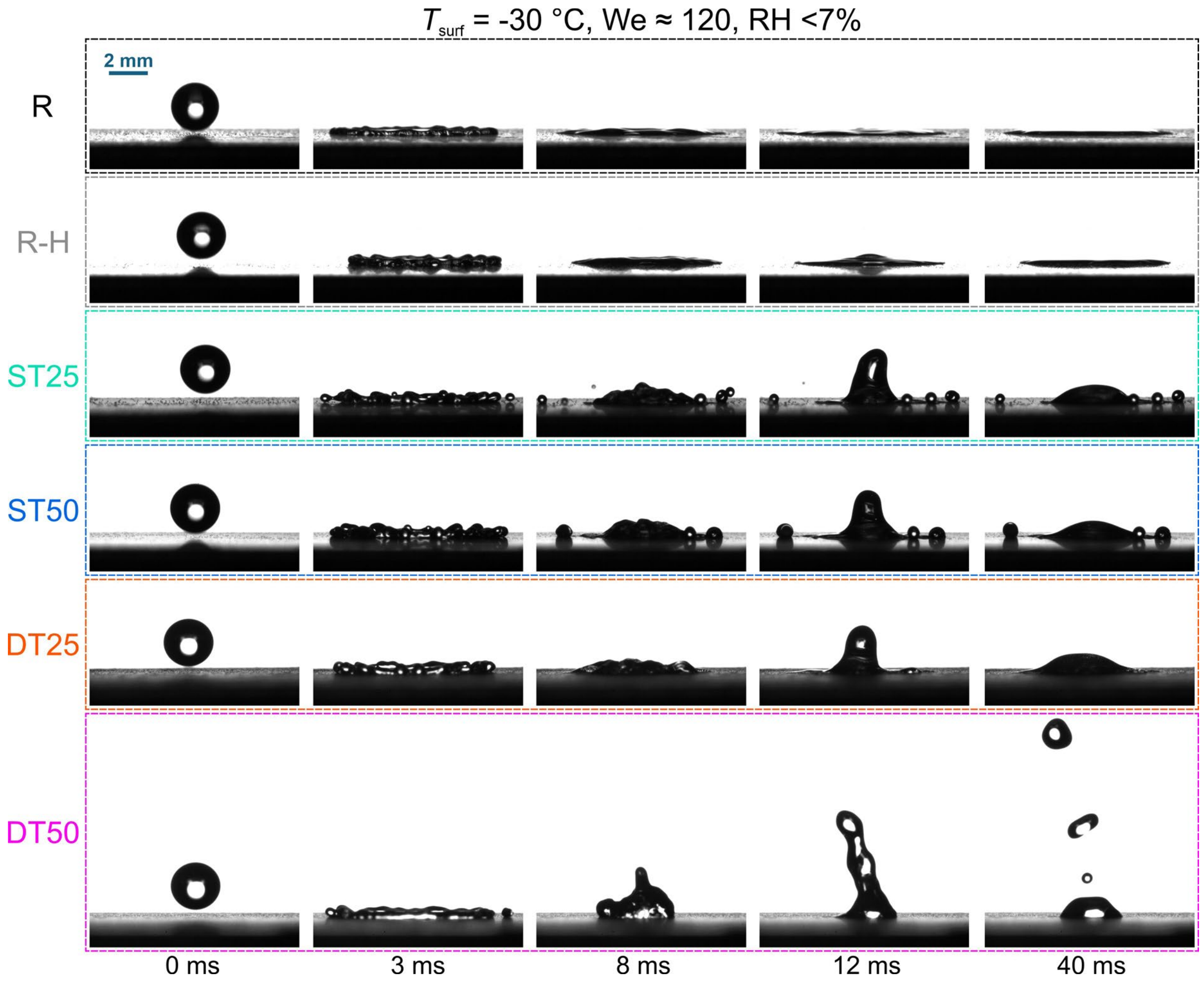

Figure S11. Droplet impacts at a surface temperature of -30 °C and We ≅ 120.

S8. Snapshots of droplet impacts at various temperatures and We ≅ 185

Figures S12-S14 show a comparison of droplet impacts at Weber number of 185 ± 8 on all six surfaces at three selected temperatures, namely 25 °C, -15 °C, and -30 °C.

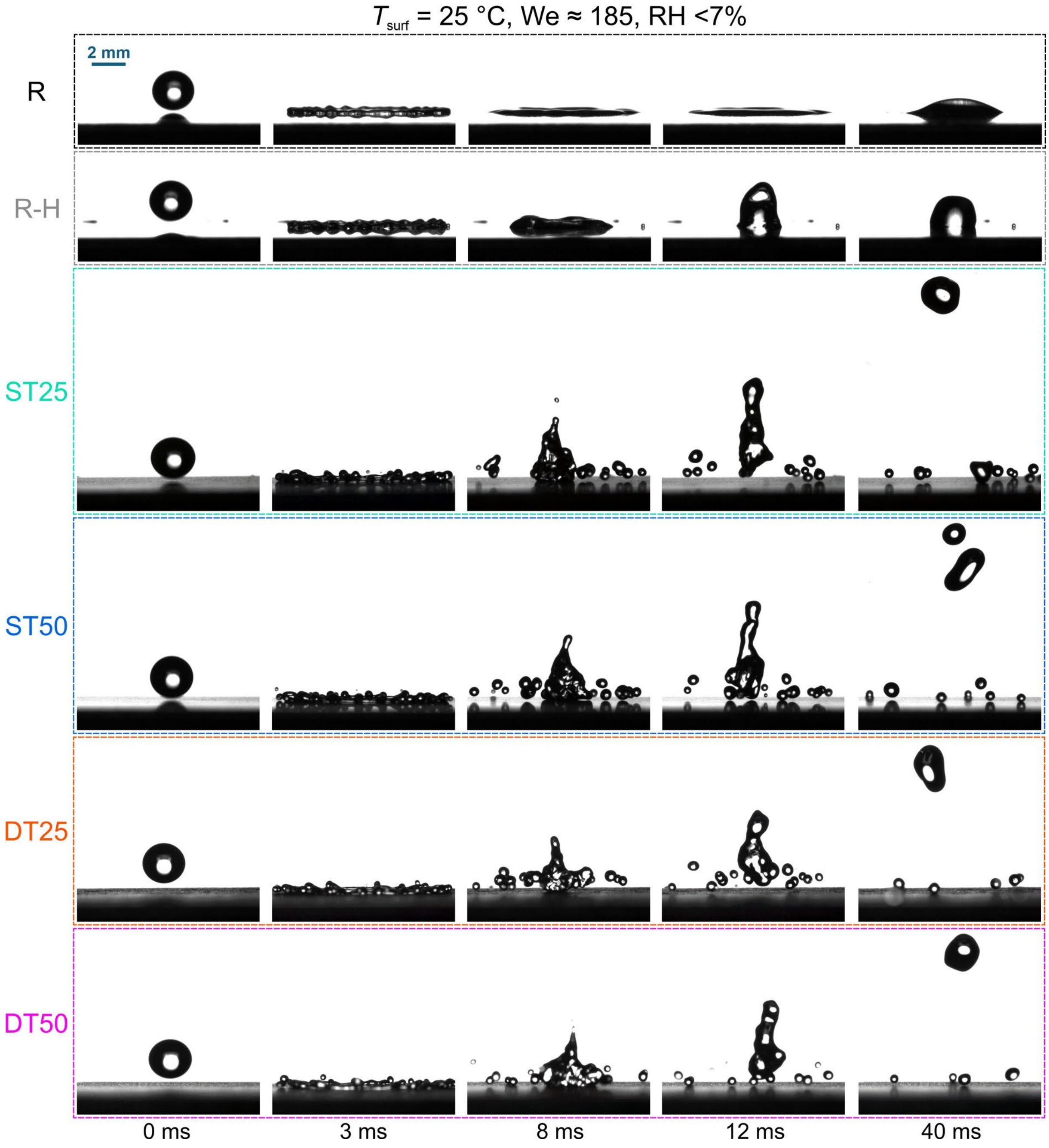

Figure S12. Droplet impacts at a surface temperature of 25 °C and We ≅ 185.

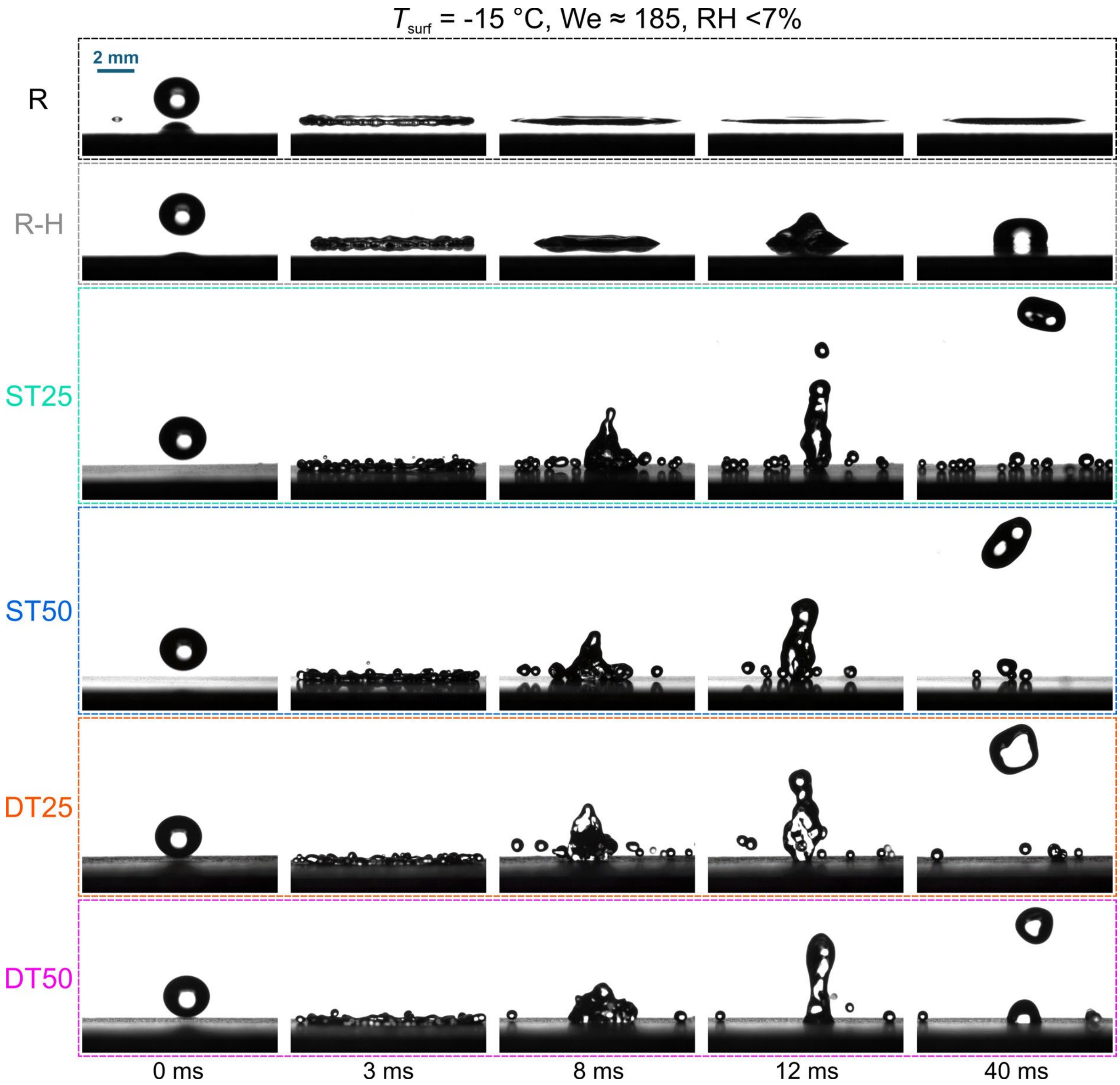

Figure S13. Droplet impacts at a surface temperature of -15 °C and We ≅ 185.

.

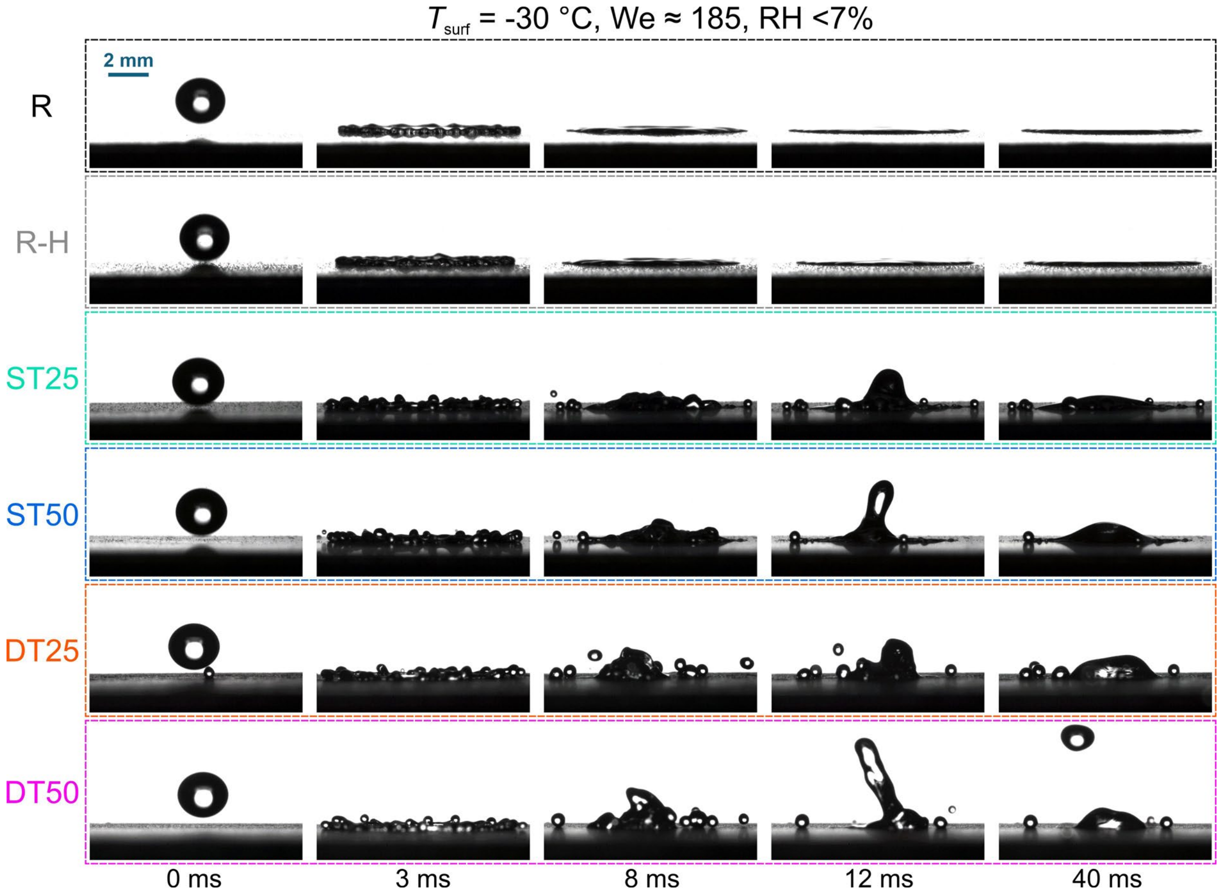

Figure S14. Droplet impacts at a surface temperature of -30 °C and We ≅ 185.

S9. One-way analysis of variance (ANOVA) for contact times at 25 °C

We performed a one-way ANOVA for the contact times for tests conducted at 25 °C to verify that the contact times are independent of surface morphology. The results are listed in Table S5. Based on the results, we can conclude that the surface morphology does not significantly affect the droplet-surface contact time as the p-value is much higher than the common significance threshold of 0.05. This statistical test should be interpreted with caution since the number of samples for each combination of testing parameters was small ($n = 3$).

Table S5. ANOVA of droplet-surface contact times at 25 °C at three different average Weber numbers for four tested surfaces.

**We**	***F*-value**	***p*-value**	**Significant?**
50	0.899	0.483	NO; $p \gg 0.05$
120	0.520	0.619	NO; $p \gg 0.05$
185	1.011	0.437	NO; $p \gg 0.05$

S10. Snapshots of droplet impacts at two different relative humidity levels

In Figs. S15-S18, snapshots of the droplet impact are compared on the ST50 and the DT25 surface for two different humidity levels. Humidity of 15-20% was achieved using silica gel, while values below 7% were recorded with the use of molecular sieves. The comparison is made for all three Weber numbers and two selected temperatures (-15 °C and -30 °C).

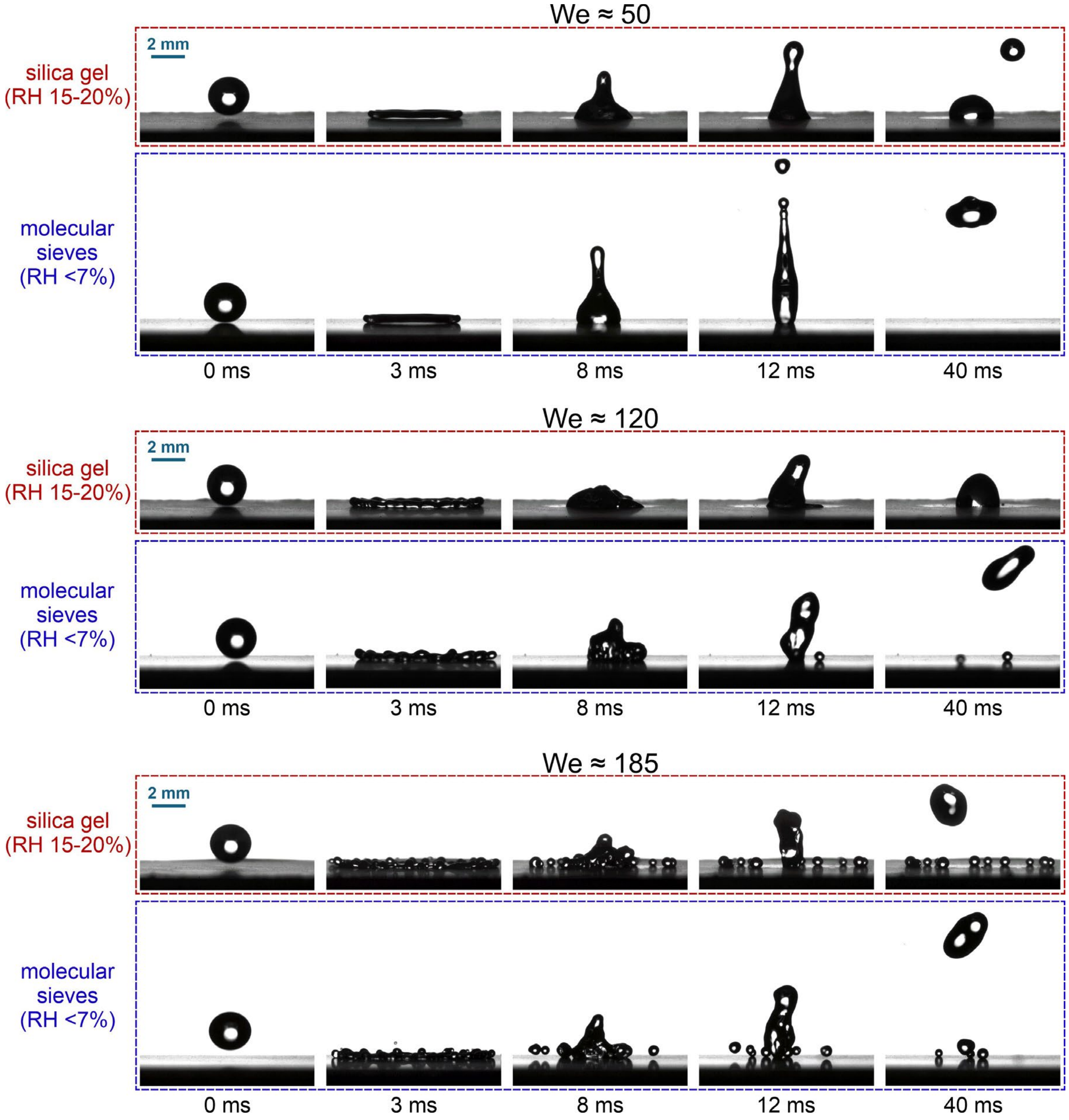

Figure S15. Droplet impacts on the ST50 surface at a surface temperature of -15 °C for two humidity levels.

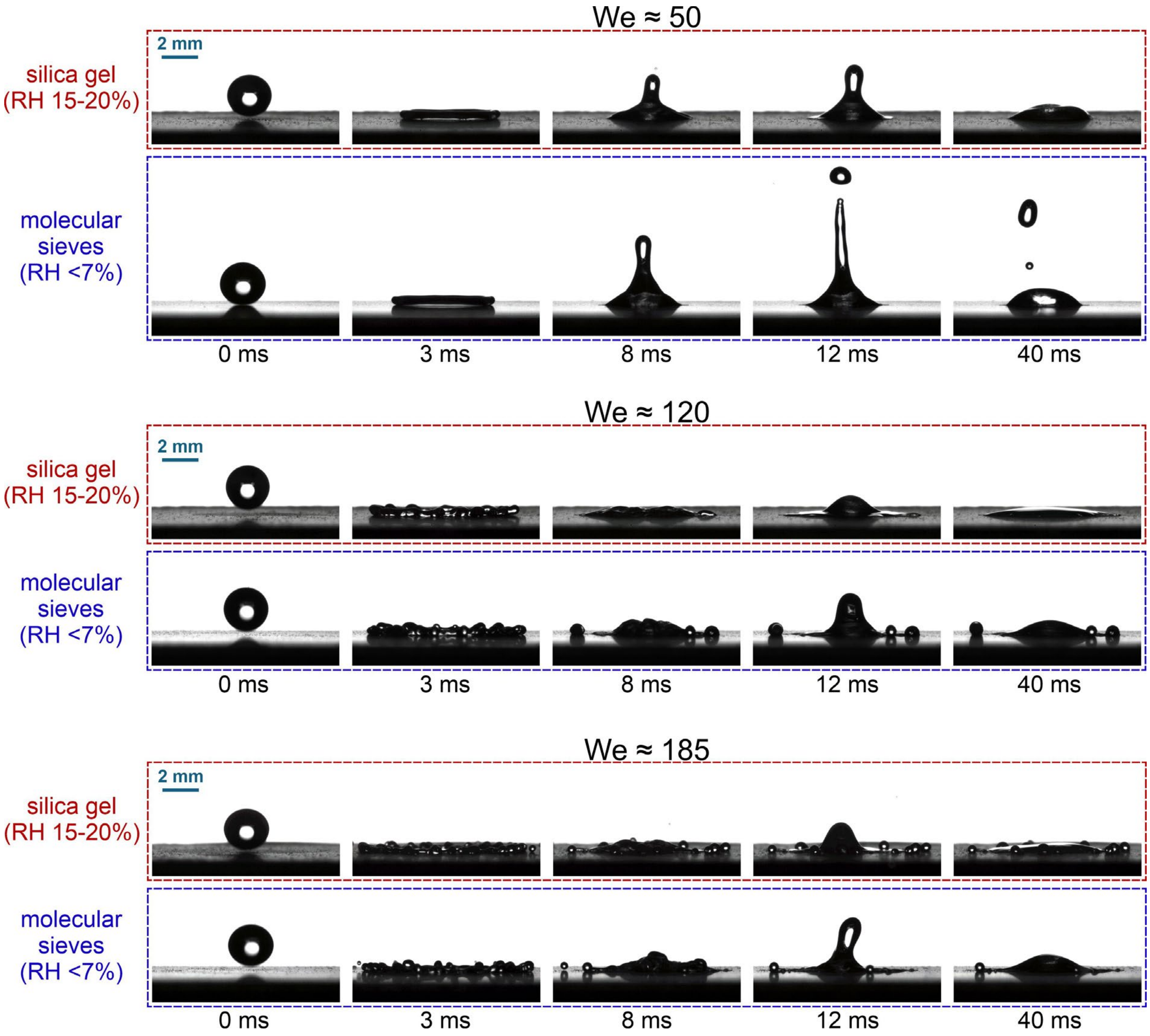

Figure S16. Droplet impacts on the ST50 surface at a surface temperature of -30 °C for two humidity levels.

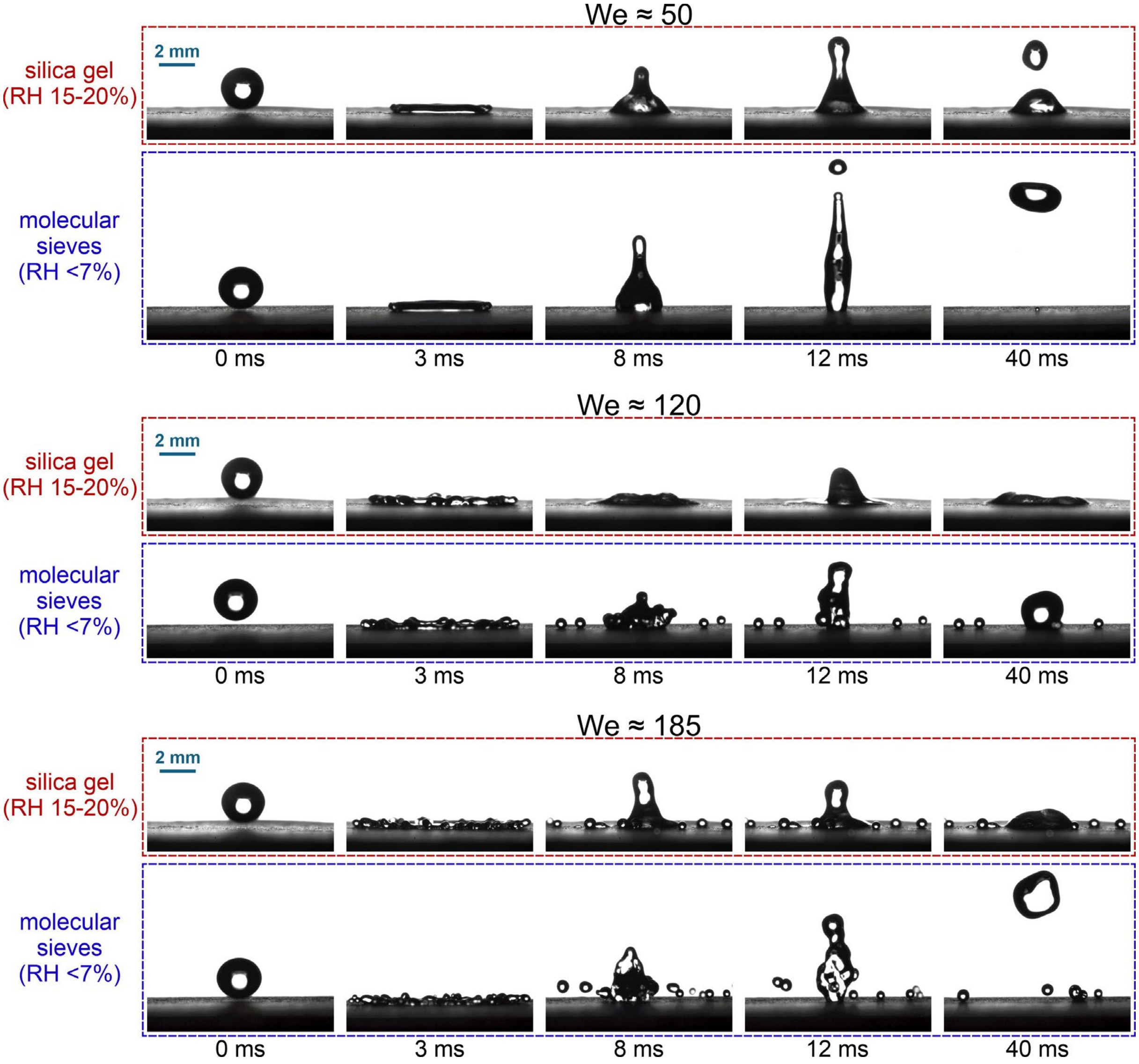

Figure S17. Droplet impacts on the DT25 surface at a surface temperature of -15 °C for two humidity levels.

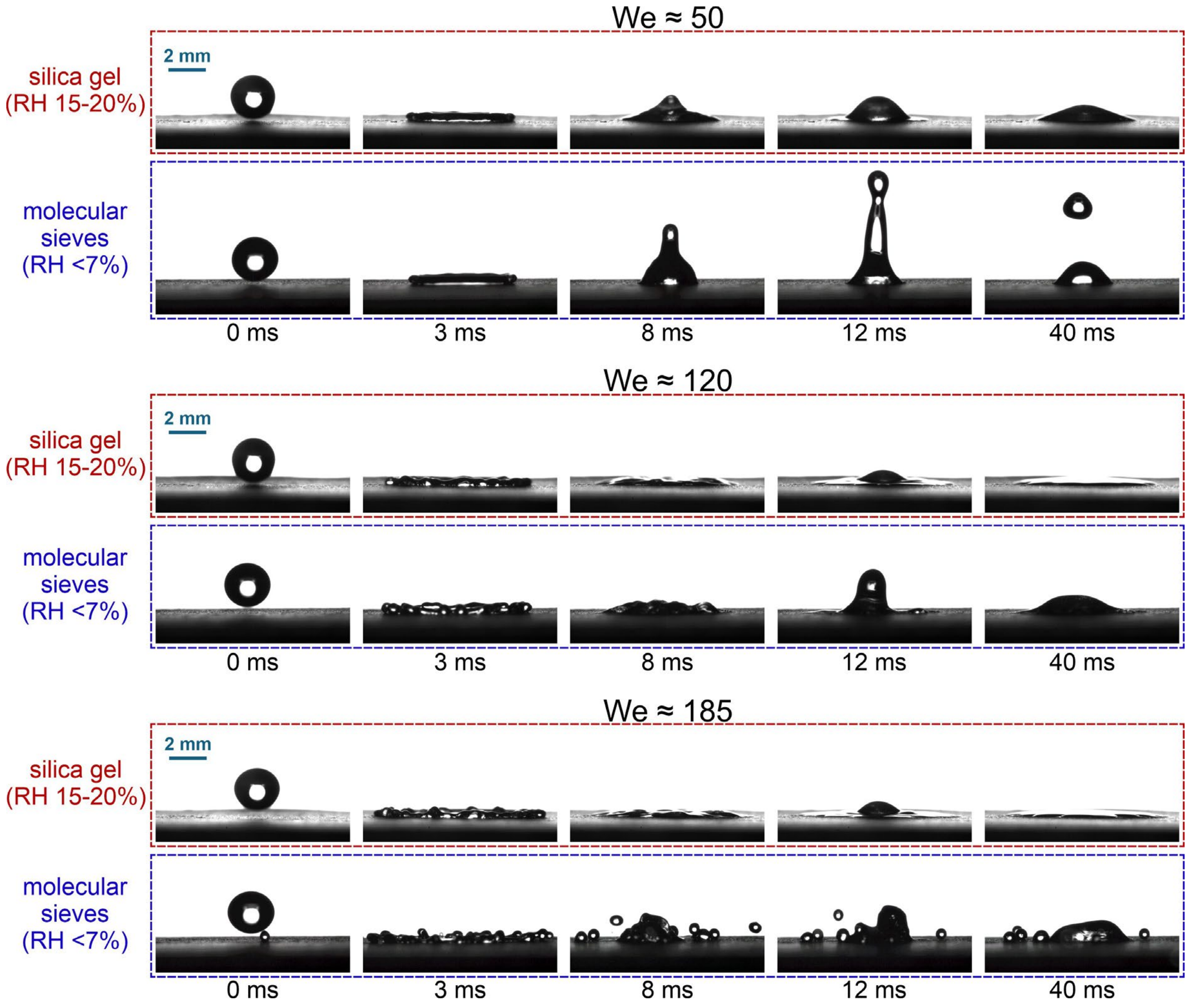

Figure S18. Droplet impacts on the DT25 surface at a surface temperature of -30 °C for two humidity levels.

References

[1] H. Jones, Cooling, freezing and substrate impact of droplets formed by rotary atomization, J. Phys. D. Appl. Phys. 4 (1971) 1657–1660. https://doi.org/10.1088/0022-3727/4/11/206.

[2] A. Asai, M. Shioya, S. Hirasawa, T. Okazaki, Impact of an ink drop on paper, J. Imaging Sci. 37 (1993) 205–207.

[3] I. V. Roisman, Inertia dominated drop collisions. II. An analytical solution of the Navier-Stokes equations for a spreading viscous film, Phys. Fluids. 21 (2009). https://doi.org/10.1063/1.3129283.

[4] B.L. Scheller, D.W. Bousfield, Newtonian drop impact with a solid surface, AIChE J. 41 (1995) 1357–1367. https://doi.org/10.1002/aic.690410602.

[5] S. Chandra, C.T. Avedisian, On the collision of a droplet with a solid surface, Proc. R. Soc. A Math. Phys. Eng. Sci. 432 (1991) 13–41. https://doi.org/10.1098/rspa.1991.0002.

[6] T. Mao, D.C.S. Kuhn, H. Tran, Spread and Rebound of Liquid Droplets upon Impact on Flat Surfaces, AIChE J. 43 (1997) 2169–2179. https://doi.org/10.1002/aic.690430903.

[7] Y.T. Aksoy, P. Eneren, E. Koos, M.R. Vetrano, Spreading of a droplet impacting on a smooth flat surface: How liquid viscosity influences the maximum spreading time and spreading ratio, Phys. Fluids. 34 (2022). https://doi.org/10.1063/5.0086050.

[8] M. Pasandideh-Fard, Y.M. Qiao, S. Chandra, J. Mostaghimi, Capillary effects during droplet impact on a solid surface, Phys. Fluids. 8 (1996) 650–659. https://doi.org/10.1063/1.868850.
